# Hidden in historical sources: Rediscovering the thorny oyster (*Spondylus gaederopus* Linnaeus, 1758) in art, archives, and cultural knowledge

**DOI:** 10.1371/journal.pone.0356273

**Published:** 2026-10-14

**Authors:** Louise Merquiol, Anne-Sophie Tribot, Daniel Faget, Thomas Changeux

**Affiliations:** 1 Aix-Marseille Univ, Université de Toulon, CNRS, IRD, MIO, Campus Technologique et Scientifique de Luminy, Marseille, France; 2 Aix-Marseille Univ, CNRS, TELEMME, Maison Méditerranéenne des Sciences Humaines et sociales, Aix-en-Provence, France; Bigelow Laboratory for Ocean Sciences, UNITED STATES OF AMERICA

## Abstract

The thorny oyster (*Spondylus gaederopus*), a Mediterranean bivalve prised during prehistoric times for its distinctive spiny purple shell, has since been overlooked in cultural and scientific records. Frequently depicted in Early Modern still-life paintings, particularly in Italian works of the seventeenth century, its representations were historically misidentified as scallops or common oysters. This participated in obscuring the visual evidence of occurrence and human use of this species during this period. Here, we adopt an interdisciplinary approach combining art historical analysis, taxonomic verification, archival research, and ethnobiological investigation to reconstruct the cultural and ecological history of *S. gaederopus* in Italy. We identify the species in 43 paintings dated between 1635 and 1750, predominantly by members of the Neapolitan Recco family, revealing its former abundance in coastal markets. Complementary historical documents, local knowledge, and oral testimonies confirm its culinary and cultural significance across Italy from the Early Modern period to the twentieth and twentieth-first century. Comparative measurements indicate a decline in average shell size since the seventeenth century, consistent with a potential harvesting pressure. Our findings demonstrate that unconventional sources including visual art, archival records, and oral testimonies can provide rich ecological and cultural insights, recovering lost biodiversity references and revealing human-marine interactions over centuries. Integrating such interdisciplinary evidence not only sheds light on the forgotten history of *S. gaederopus* but also highlights the potential of combining cultural heritage and scientific research to inform contemporary conservation strategies.

## Introduction

The thorny oyster or spiny oyster (*Spondylus gaederopus* Linnaeus, 1758) is an endemic bivalve mollusc spread across the Mediterranean Sea (Fig 1A). Although commonly referred to as an oyster, it is phylogenetically distinct from true oysters (family Ostreidae) and instead belongs to the superfamily Pectinoidea, which also includes scallops (family Pectinidae). The species typically inhabits hard substrates such as rocky outcrops and coralligenous assemblages at depths of a few to about 50 m, as indicated in general identification guides. With an average diameter of 60–125 mm [1], *S. gaederopus* generally exceeds the size of the European flat oyster (*Ostrea edulis*). It is characterised by a thick, inequivalve shell with the lower (right) valve cemented to the substrate and an articulated upper (left) valve bearing prominent radial spines often encrusted with epibionts, particularly the red sponge *Crambe crambe*, which provide effective camouflage in natural settings (Fig 1B-C). When cleaned of epibionts, the upper valve reveals vivid pigmentation ranging from violet and carmine to red-purple, a coloration that earned it the vernacular misnomer ‘*imperial oyster*’ (*‘ostrica imperiale’* in Italian) despite its closer taxonomic affinity to scallops. Ecologically, *S. gaederopus* is a long-lived, slow-growth species with a strong substrate specificity, which heightens its potential vulnerability to environmental change and anthropogenic disturbance.

**Fig 1:**
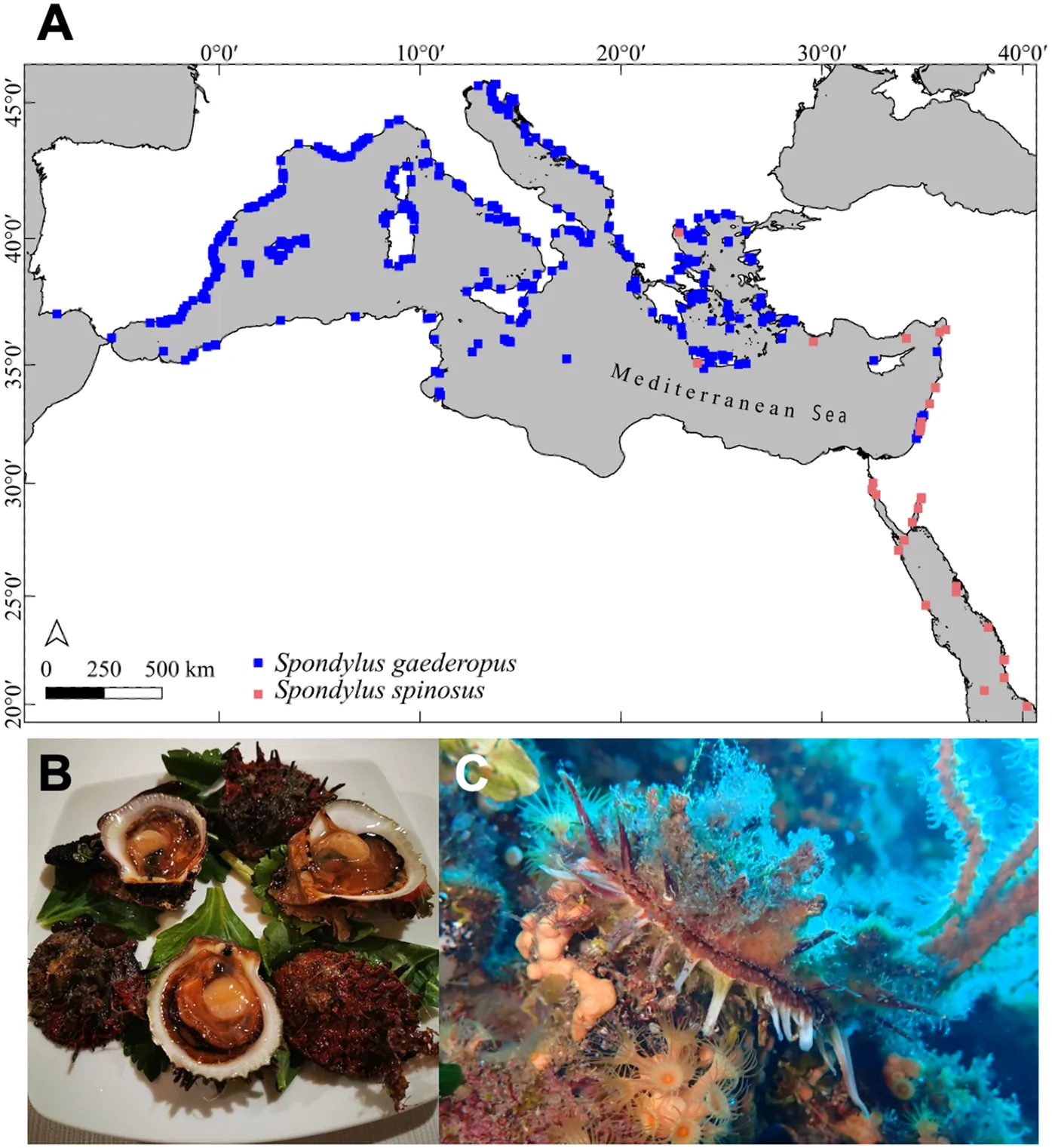
Current distribution and field records of *Spondylus gaederopus* and *S. spinosus* in the Mediterranean Sea. **A.** Occurrences of the endemic *Spondylus gaederopus* and the invasive *S. spinosus* in the Mediterranean Sea. GBIF occurrence datasets https://doi.org/10.15468/dl.4u6bru (*S. gaederopus*) and https://doi.org/10.15468/dl.h6nync (*S. spinosus*) accessed via GBIF.org (08/09/2025), map made on QGIS with NaturalEarth [free vector and raster map data @naturalearthdata.com, public domain]. **B.** Photography of *S. gaederopus* taken by a restaurant customer in Apulia (south Italy) in January 2020 (photo credits: Tripadvisor). **C.** Photography of *S. gaederopus* taken in September 2025 in a rocky outcrop in Ile Jarre, Western Mediterranean Sea (photo credits: Sylvain Le Bris).

The distinctive morphological features of thorny oysters, combined with their accessibility in shallow waters, have fostered a long history of human utilization. Archaeological evidence underscores their cultural significance across regions, including pre-Columbian America and the Mediterranean, where *Spondylus* shells were crafted into beads, pendants, and other ornaments [2,3]. In Europe, such artifacts have been recovered from inland Neolithic sites far from coastal areas, reflecting extensive trade networks and the species’ high symbolic and economic value in prehistoric societies [4,5]. The Adriatic region emerged as a key production centre for the manufacture of *S. gaederopus* jewellery [6], as attested by substantial quantities of shells dating from 6000−4700 BCE discovered throughout the Italian peninsula [7]. While primarily used for ornamental and utilitarian purposes, archaeological deposits confirm that *S. gaederopus* was also consumed as food in Spain [8] and Greece [9]. Historical records further document its culinary use during Roman times, such as feasts in Brindisi [10], and its presence in the Herculaneum excavations (79 CE) highlights its role in Roman diet and trade [11]. At this period, local Greek fishermen likely employed rudimentary dredging techniques, such as long poles fitted with flat iron tips to dislodge the molluscs, which were then retrieved from the sea using an iron hand attached to the opposite end of the pole [12].

Despite its deep-rooted cultural importance in Mediterranean and European prehistoric societies, *S. gaederopus* has received limited scientific attention in recent centuries. Once common throughout the Mediterranean, the species underwent severe population declines documented from the early 1980s, when extensive and unexplained mass mortality events were reported from Greece to Spain and Algeria [see 13, 14]. Observations from French scuba divers between 1981 and 1983 indicated mortality rates exceeding 90% [13]. Similar episodic die-offs recurred during the 2000s in parts of Spain and Greece [15,16] and continue sporadically today, potentially linked to marine heatwaves or disease outbreaks [17].

Quantitative ecological assessments of *S. gaederopus* remain scarce. The only comprehensive modern study, conducted in the North Aegean Sea in 2008, documented low population densities but generally healthy individuals, with a mean shell length of 6.6 cm and an estimated maximum age of 18 years for a specimen measuring 6.9 cm [15]. Growth analyses revealed a mean annual increment of approximately 2.4 mm, consistent with the species’ slow-growing, long-lived biology. More recent size records across the Mediterranean report average shell lengths ranging from 6 to 12 cm [1].

Although *S. gaederopus* remains endemic to the Mediterranean Sea and continues to be recorded throughout the basin (Fig 1), its current conservation status is poorly defined. The species has not been evaluated by the International Union for Conservation of Nature (IUCN) and is not listed under the Convention on International Trade in Endangered Species (CITES). In the eastern Mediterranean, *S. gaederopus* notably faces increasing competition from the invasive *Spondylus spinosus* [18,19], a Lessepsian migrant first recorded in the late 1980s off Israel [18] and successively along the Cypriot [20] and Turkish coasts [21] (Fig 1A). This species has since replaced *S. gaederopus* along parts of the central Israeli coastline but has itself been affected by recent marine heatwaves [22]. Overall, the ecological trajectory of *S. gaederopus* since the Early Modern Period remains insufficiently documented, underscoring the need for updated population surveys and conservation assessments.

The limited and fragmented ecological data available for *S. gaederopus* highlight the broader challenge of reconstructing the historical dynamics of Mediterranean marine species. To address these limitations, interdisciplinary approaches that combine biological evidence with historical and ethnographic records can provide a more comprehensive understanding of species trajectories through time. In the absence of consistent and continuous scientific monitoring, unconventional sources serve as important repositories of biodiversity information and human-species interactions. Historical and ethnographical evidence, including written documents, pictorial representations, oral testimonies, and local knowledge, have proven critical for reconstructing socio-ecological systems and informing biodiversity research [23]. Such integrative frameworks have already yielded important insights into past biodiversity change [24] and the recovery of inconspicuous species [25]. This approach is particularly relevant for marine environments, where ecological and cultural records and often fragmented and temporally discontinuous. Integrating these diverse unconventional sources with more conventional ones can therefore offer powerful means of recovering lost ecological and cultural information.

Among these sources, visual arts – especially still-life paintings – play a distinctive role in documenting species with remarkable fidelity. Such artworks not only record morphological details but also reflect aspects of everyday life, trade, and consumption [25,26]. In the present study, we combine the analysis of Early Modern (sixteenth-eighteenth centuries) Italian still-life paintings with complementary historical and ethnographic sources to trace the cultural and ecological history of *S. gaederopus*. Specifically, we explore how unconventional materials can function as historical-ecological tools to reconstruct the use, perception, and consumption of species that have otherwise faded from both scientific and cultural memory. Our objectives are to i) identify representations of *S. gaederopus* in Early Modern Italian still-life paintings, ii) assess patterns of depiction across geographic and temporal contexts, and iii) recover from historical evidence the use and cultural significance of the species from the Early Modern period to the present. By integrating art history, taxonomy, and ethnobiology, this study seeks to recover a more comprehensive understanding of human-nature interactions and to reconsider the cultural afterlives of forgotten species.

## Materials and methods

### Historical evidence of *S. gaederopus* in pre-modern and modern Italy

To contextualize the historical and contemporary presence of *S. gaederopus* in Italy, we reviewed both archival and ethnographic sources. Sources encompassed ecological inventories, historical and cultural writings, pictorial representations, online accounts of local knowledge, and oral testimonies. Special attention was given to records documenting shell consumption, connoisseurship, and Mediterranean trade networks involving molluscs.

Within the pictorial sources, we reviewed a corpus of 365 Italian still-life paintings produced between the sixteenth and eighteenth centuries and depicting marine organisms. These paintings were sourced from a previously established dataset, for which the collection protocol is detailed in Merquiol et al. (2025). Among them, 127 paintings were identified as containing representations of fresh marine molluscs and ascidians (S1 Table). High-resolution digital reproductions were examined to allow detailed visual inspection and taxonomic identification.

Fresh marine molluscs and ascidians were identified based on morphological characteristics observable in the paintings, including shape, coloration, ribbing, spines and hinge details. These visual features were systematically compared with those of physical specimens to support accurate identification. Particular attention was given to distinguishing the thorny oyster (*S. gaederopus*) from other visually similar invertebrates frequently depicted in art, such as the great scallop (*Pecten maximus*), true oysters (*Ostrea* spp.), limpets (*Patella* spp.), and edible ascidians (*Microcosmus sabatieri*). Edible ascidians, commonly known as sea figs, were also considered in this analysis due to their fleshy, lobed bodies encased in a leathery tunic that, when opened, strongly resembles molluscs. Key diagnostic features that we used to identify *S. gaederopus* included the presence of a black ligament, a distinctive hinge structure, external supination with epibionts, and an internal orange-yellow coloration with a large central muscle typical of Pectinoidea (Fig 2). Other bivalves were recognized by their characteristic morphologies, such as the pear-shaped, rough-surface of oysters, the fan-shaped shell with radial ribs of scallops, and the conical shell with blue margins typical of limpets.

**Fig 2:**
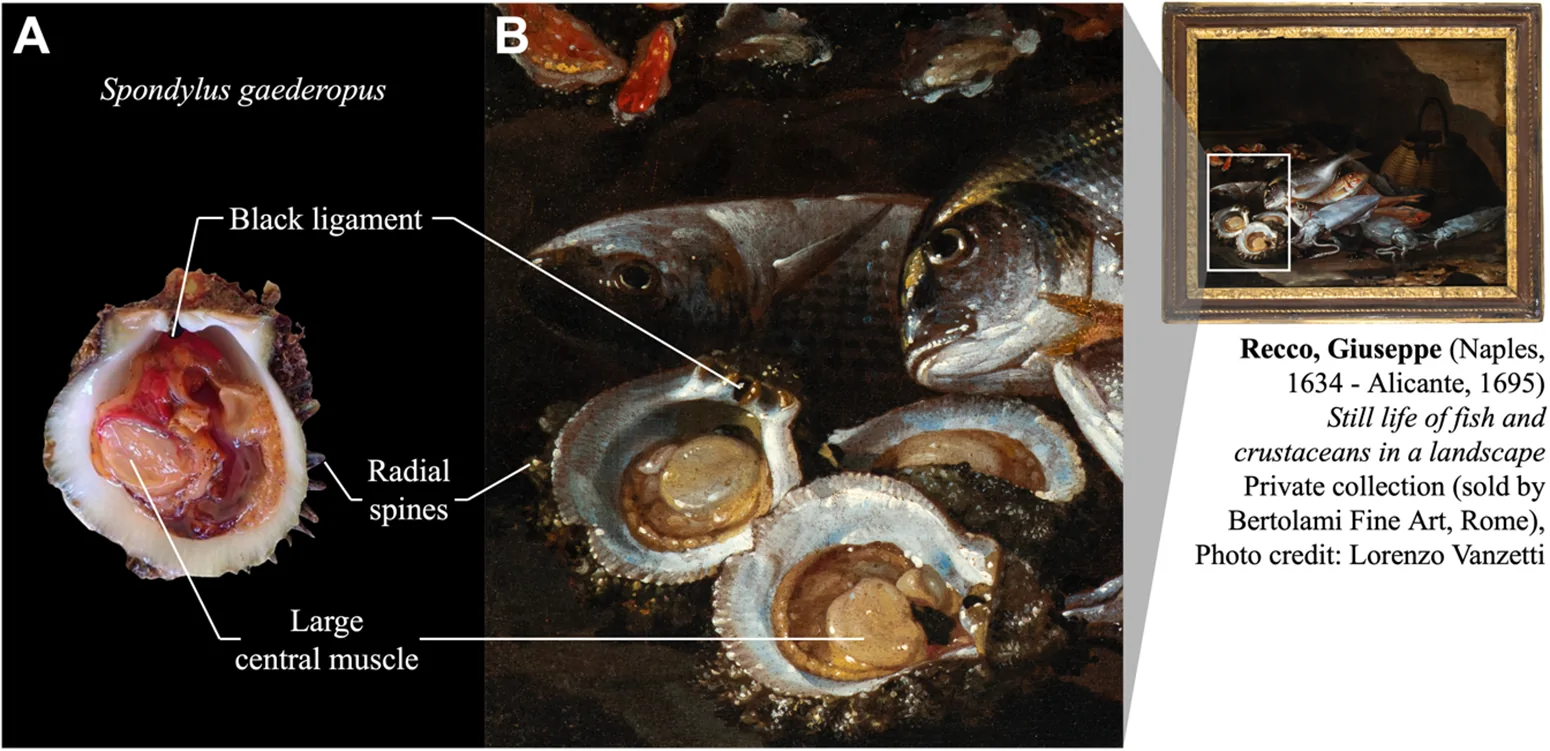
Anatomical characteristics of *Spondylus gaederopus.* **A** Photo of *Spondylus gaederopus* with anatomical details (photo credits: Tripadvisor) and **B** Oil on canvas by Giuseppe Recco (Naples, 1634 - Alicante, 1695), *Still life of fish and crustaceans in a landscape*, 71.5 × 64 cm [private collection, Naples], where three specimen of *S. gaederopus* were identified with the same anatomical details (image courtesy of Bertolami Fine Art, photo credits: Lorenzo Vanzetti, all rights reserved).

The visual identification also considered the other co-occurring marine species within each painting. All organisms were identified at the lowest taxonomic level by taxonomic experts following the INPN taxonomic reference [27]. A total of 97 marine and diadromous taxa from Mediterranean and North Atlantic origin were determined, excluding empty shells (S2 Table). The presence of accessories, such as baskets, copper pots, fishing gears, buckets, plates, and cooking ingredients, was also accounted for.

### Estimated sizes of molluscs

A quantitative component was integrated into this study by measuring the total lengths of represented molluscs in paintings. In addition, *S. gaederopus* specimens were also measured from online contemporary photographs (searched using the keywords *‘ostrica imperiale’* and *‘ostriche imperiali’* on Facebook.com). Measurements were estimated using reference scales available within each image, such as the average diameter of mature sea urchin *Paracentrotus lividus* (approximately 6 cm) or the average length of human finger metacarpals, providing a basis for relative size assessment. Although the accuracy of measurements was constrained by the limited availability of reliable comparative reference elements, a total of 84 molluscs were measured in 19 still-life paintings (S3 Table). A Student test was performed on normalized data to compare *S. gaederopus* shell sizes between media (*t.*test from the package *stats* on R).

### Local knowledge and oral testimony

Contemporary references were also investigated online to document recent knowledge and practices associated with *S. gaederopus* in Italy. An online article from Naples specifically discussing the species was identified by the authors [28]. The author of the article was contacted and agreed to participate in an interview aimed at further exploring the local knowledge underlying his observations. Based on information provided during the exchange, he appeared to have extensive experience with local marine environments and fishing activities. The semi-structured interview focused on the local occurrence, recognition, collection, consumption, and vernacular knowledge of *S. gaederopus*, as well as changes observed over time.

To complement this oral testimony, contemporary online references were systematically searched for additional evidence of local knowledge and recent observations. Searches included publicly available user-generated content on platforms such as Facebook and Tripadvisor, using combinations of the scientific name, vernacular names (including *‘ostrica imperiale’* and *‘ostriche imperiali’*), and geographically specific terms. These sources were used to identify recent observations, descriptions of the species, accounts of its consumption or collection, and references to its local availability. Online accounts were treated as complementary qualitative sources rather than as systematic ecological observations, and were used primarily to contextualize the historical records and assess the persistence of local knowledge concerning *S. gaederopus*.

## Results

### Identification of *S. gaederopus* in Early Modern still-life paintings

Searches in our database using vernacular names of *S. gaederopus* (in English and in Italian) yielded no direct matches in the titles of Early Modern paintings. Nonetheless, based on the species’ distinctive morphological features, we confidently identified the species in 43 artworks (Fig 3). Titles assigned by art historians rarely specified the marine species depicted. Some paintings omitted any mention of shellfish altogether, such as *Natura morta con pesci* (Nicola Maria Recco), while others employed only general descriptions, for example *Still life with fish and molluscs* (Giuseppe Recco). In several cases, the species was also misidentified. For instance, Giovanni Battista Recco’s *Panier d’huîtres, coquilles Saint-Jacques [scallops], oursins et couteaux sur un entablement de pierre* and *Nature morte aux poissons de mer et aux huîtres orientales [oriental oysters]* were catalogued respectively as featuring scallops (*Pecten maximus*) and “oriental oysters”, both incorrect attributions.

**Fig 3:**
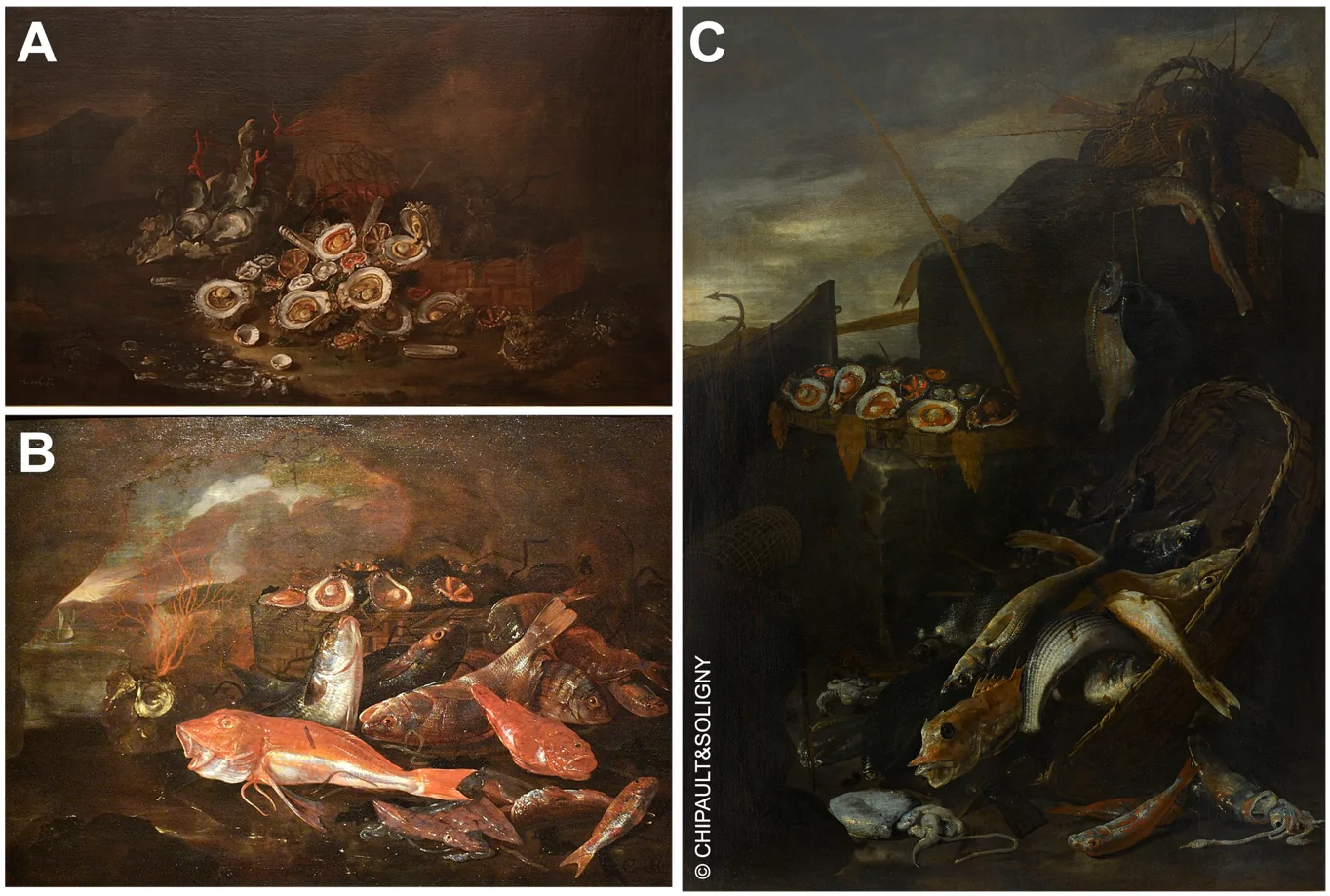
Artistic depictions of *Spondylus gaederopus* in Italian still-life paintings. **A.** Oil on canvas by Loth Onofrio (Naples, 1665 – Naples, 1715), Natura morta con corallo e ostriche (ca. 1690) [Museu de Belles Arts de València, Spain], CC-BY 4.0 (photo credits: Francesco Bini), **B.** Oil on canvas by Giuseppe Recco (Naples, 1634 – Alicante, 1695), Calamari e ostriche (ca. 1654-1695) [Museo di Capodimonte, Italy], CC-BY 4.0 (photo credits: Giuseppe Guida), **C.** Oil on canvas by Giovanni Battista Recco (Naples, 1615 – Naples, 1660), Nature morte aux poissons de mer et aux huîtres orientales (ca. 1655-1660) [Musée des Beaux-Arts et d’Archéologie, Besançon, France] (image courtesy of Musée des Beaux-Arts et d’Archéologie de Besançon, photo credits: Chipault & Soligny, all rights reserved).

All identified paintings were produced between 1635 and 1750 by 13 artists, the majority of whom were active in Naples (Fig 4A). Depictions of *S. gaederopus* were particularly concentrated within the Neapolitan Recco family of painters. Together, Giuseppe Recco (Naples, 1634 – Alicante, 1695), his son Nicola Maria Recco (Naples, early eighteenth century) and his brother Giovanni Battista Recco (Naples, 1615 – Naples, 1660), accounted for 30 of the 43 identified paintings. Giuseppe Recco alone depicted *S. gaederopus* in 14 of his 18 attributed still-lifes, followed by Nicola Maria in 10 of 11, and Giovanni Battista in 6 of 7 works (Fig 4B). No clear temporal or artist-specific variation was observed in the depiction of *S. gaederopus*. The number of specimens represented per painting ranged from one to eight, with the highest counts occurring from 1670 (see S1 Fig). However, this apparent consistency may reflect the relatively limited number of artworks available for analysis.

**Fig 4:**
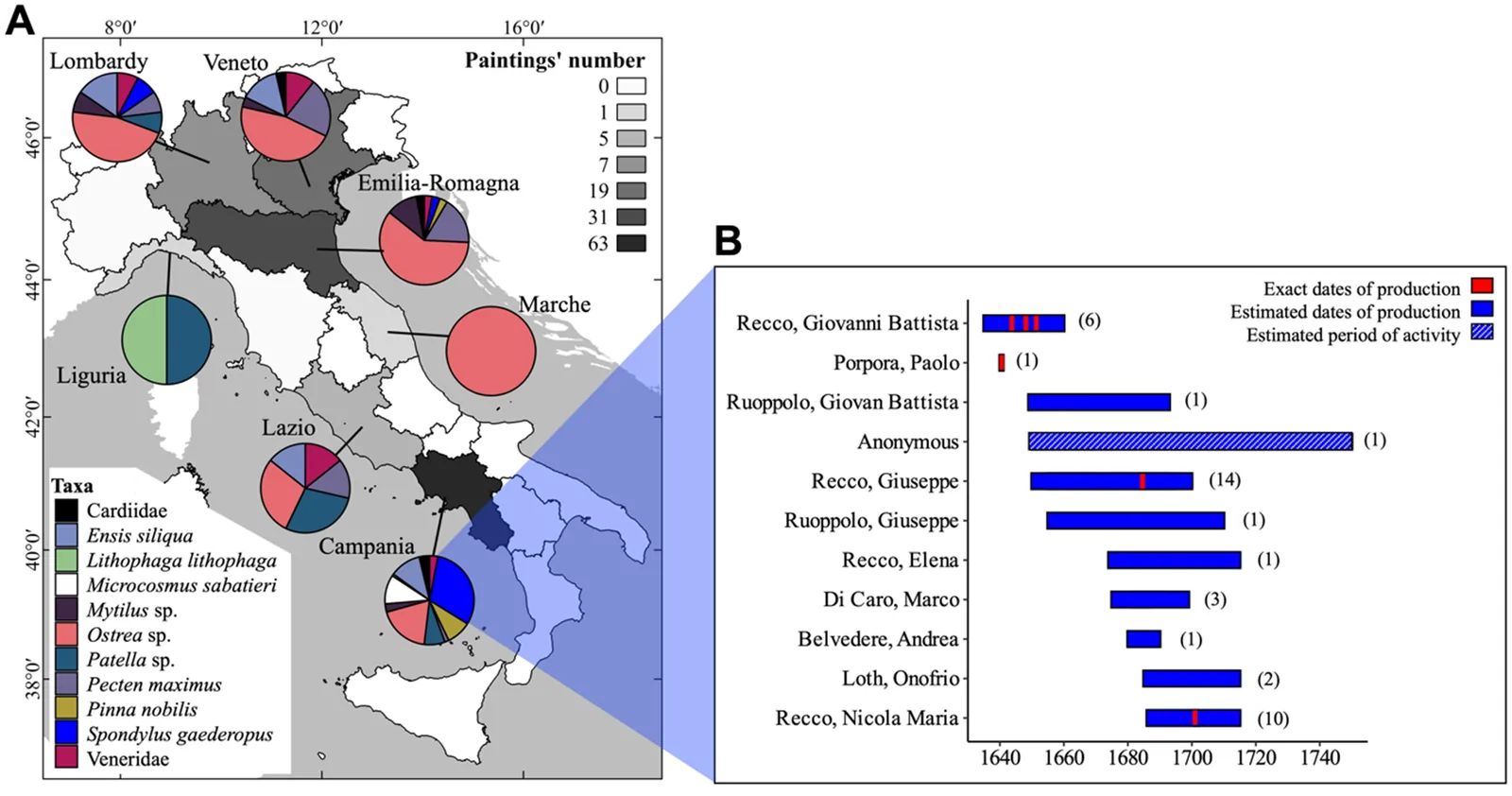
Geographical pattern of molluscs and ascidians in Italian still-life paintings with a focus on Naples. **A** Representations of marine molluscs and ascidians (in proportions) in Italian still-life paintings according to the known geographical location of artists (see https://research.rkd.nl/). Paintings were grouped by regions and the number of paintings within each region is indicated by the grey scale, made on QGIS with NaturalEarth [free vector and raster map data @naturalearthdata.com, public domain]; **B** Dates of production of paintings representing *S. gaederopus* by Neapolitan painters. Numbers in parenthesis indicate the number of paintings produced by each painter.

### Associations of taxa alongside *S. gaederopus*

Across the 127 examined still-life paintings, morphological analysis allowed to identify ten mollusc taxa (bivalves and gastropods) and one ascidian species (Table 1). Oysters (*Ostrea* spp.) were by far the most frequently depicted, appearing in about 53% of paintings, followed by *S. gaederopus* in 34%, and in nearly two-thirds of works produced in Campania. Razor shells (*Ensis siliqua*) were less common (17%), while scallops (*Pecten maximus*), sea figs (*M. sabatieri*), limpets (*Patella* spp.), and noble pen shells (*Pinna nobilis*) appeared only occasionally. Most taxa represented were, and remain, traditional components of Mediterranean coastal diets. Regional variation was marked across the seven Italian regions represented in the dataset. Painters from Adriatic regions depicted oysters most frequently (46%−100%), whereas artists from Campania showed greater taxonomic diversity, representing all identified taxa (Fig 4A).

**Table 1:** Number and proportions of Italian still-life paintings from the sixteenth-eighteenth centuries depicting fresh marine bivalves, gastropods, and ascidians, based on a total of 127 analysed works.

Identified taxa	Number of paintings	Percentage
**Bivalves**		
*Ostrea* spp.	68	53.5%
*Spondylus gaederopus*	43	33.9%
*Ensis siliqua*	22	17.3%
*Pecten maximus*	16	12.6%
*Pinna nobilis*	13	10.2%
*Mytilus* spp.	10	7.9%
Veneridae	10	7.9%
Cardiidae	7	5.5%
*Lithophaga lithophaga*	2	1.6%
**Gastropods**		
*Patella* spp.	14	11.0%
**Ascidians**		
*Microcosmus sabatieri*	14	11.0%
**Total**	**127**	**–**

Italian still-life painters often depicted molluscs and ascidians within rich assemblages of other marine organisms (Fig 5). The most frequently represented taxa were sea urchins (*Paracentrotus lividus*), often shown opened and ready for consumption. They were commonly accompanied by commercially and culturally important fish such as mullets (Mugilidae), goatfishes (*Mullus* spp.), and salemas (*Sarpa salpa*), as well as cephalopods like squids (*Loligo* spp.). In these paintings, *S. gaederopus* was recurrently portrayed alongside species typical of Mediterranean rocky shores, including sea urchins, spiny lobsters (*Palinurus elephas*), scorpionfishes (Scorpaenidae), seabreams (*Diplodus* spp.), and oysters (see examples in Fig 3).

**Fig 5:**
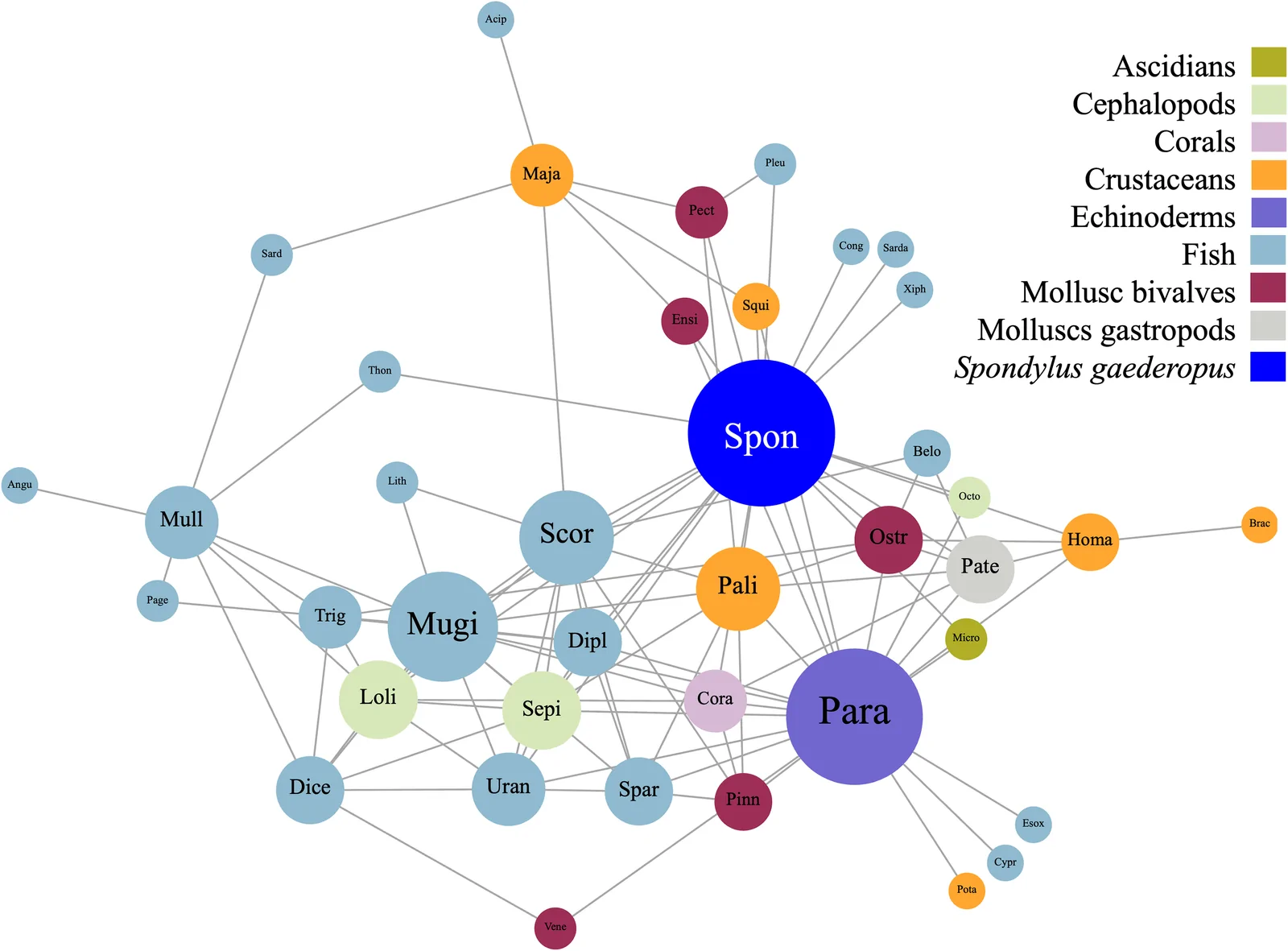
Aquatic taxa co-occurrence network in Italian still-life paintings. Node sizes indicate the number of taxonomic associations, while edges reflects the co-occurrences within paintings. Abbreviations: Acip, *Acipenser sturio*; Angu, *Anguilla anguilla*; Belo, *Belone spp.*; Brac, *Brachyura*; Cong, *Conger conger*; Cora, *Corallium rubrum*; Cypr, *Cyprinus carpio*; Dice, *Dicentrarchus labrax*; Dipl, *Diplodus spp.*; Ensi, *Ensis siliqua*; Esox, *Esox spp.*; Homa, *Homarus gammarus*; Lith, *Lithognathus mormyrus*; Loli, *Loligo spp.*; Maja, *Maja sp.*; Micro, *Microcosmus sabatieri*; Mugi, *Mugilidae*; Mull, *Mullus spp.*; Octo, *Octopus vulgaris*; Ostr, *Ostrea spp.*; Page, *Pagellus spp.*; Pali, *Palinurus elephas*; Para, *Paracentrotus lividus*; Pate, *Patella spp.*; Pect, *Pecten maximus*; Pinn, *Pinna nobilis*; Pleu, *Pleuronectidae*; Pota, *Potamon fluviatile*; Sarda, *Sarda sarda*; Sard, *Sardina pilchardus*; Scor, *Scorpaenidae*; Sepi, *Sepia officinalis*; Spar, *Sparus spp.*; Squi, *Squilla mantis*; Spon, *Spondylus gaederopus*; Thon, *Thunnus spp.*; Trig, *Triglidae*; Ura, *Uranoscopus scaber*; Vene, *Veneridae*; Xip, *Xiphias gladius*.

### Sizes measurements of molluscs

Size measurements of molluscs were constrained by the one-dimensional nature of the painted representations. Nevertheless, reliable mean length estimates were obtained from 19 paintings for *S. gaederopus* (8.5 ± 1.0 cm), *Patella* sp. (5.0 ± 1.3 cm), *Ostrea* sp. (6.2 ± 2.0 cm), *P. nobilis* (34.2 cm), *E. siliqua* (8.7 ± 1.2 cm), and *Lithophaga lithophaga* (7.7 ± 0.4 cm) (Fig 6).

**Fig 6:**
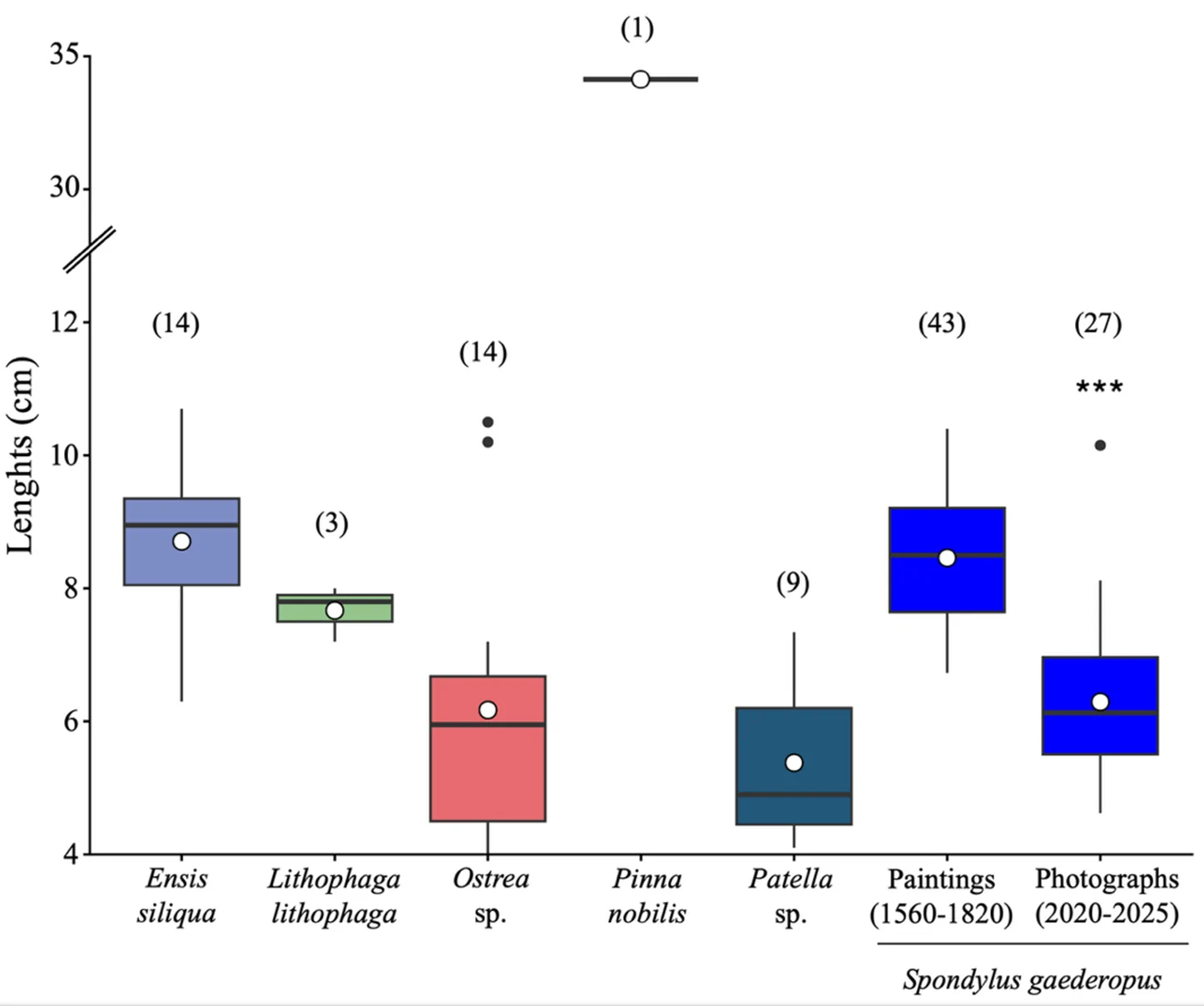
Size measurements of molluscs performed on 19 paintings. Only size differences of *S. gaederopus* were compared between historical paintings and modern photographs. The boxplots display the interquartile range (boxes), median (horizontal line), and mean (dot), with whiskers representing one standard deviation. Numbers in parentheses indicate the number of measured specimens. *** marks a statistically significant difference as determined by a t-test on normalized data (t = 7.68, df = 47.04, p < 0.001).

Measurements of *S. gaederopus* specimens indicate that individuals depicted in Early Modern still-life paintings were, on average, significantly larger (mean estimated length: 8.5 cm) than those observed in contemporary photographs taken between 2020 and 2025 (mean length: 6.3 cm) (Fig 6). This difference may reflect either an artistic preference for representing larger specimens or a historical shift in the species’ size distribution. However, these findings must be interpreted cautiously. Unlike modern photographs, which allow for direct measurement, size estimates in paintings rely on reference scales derived from accompanying objects or other depicted species, which may introduce variability. Furthermore, while reference scales were selected in close proximity to the measured specimens, potential perspective distortions could further influence the accuracy of these estimates.

### Naturalistic and historic writings, local knowledge and oral testimony on *S. gaederopus*

#### Naturalistic and historic writings.

Historical accounts from the eighteenth and nineteenth centuries consistently document the presence, collection, and occasional consumption of *S. gaederopus* along several Italian coasts. Technical manuals [29] and regional dialect dictionaries [30] likewise preserve a rich array of vernacular names for the species across Italy, as summarized in Table 2. Throughout much of the Mediterranean, and especially along the southern Italian coastline, the shell of *S. gaederopus*, frequently encrusted with the bright red sponge *Crambe crambe*, appears to have inspired several regional names, such as *‘ostriche russe’* (“red oysters”) in Salento and ‘*grattapuetoli’* (“finger-scratchers”) in the Brindisi dialect, the latter referring to the irritation caused by the sponge covering. The persistence of these diverse local names attests to the mollusc’s regional recognition despite its limited economic role. Some terminological confusion, however, has arisen with the term *‘piede d’asino’* (“donkey’s foot”), properly referring to the bittersweet clam *Glycymeris glycymeris*, which was occasionally misapplied to *S. gaederopus* in historical texts [31,32].

**Table 2:** Vernacular names historically and currently used for *Spondylus gaederopus* in Italy, including regional and dialectal variants.

Origin	Vernacular name
Italian	*Spondilo*
	*Ostrica imperiale* (plural: *ostriche imperiali*)
Italian (Adriatic)	*Cernieruolo*
Brindisi	*Grattapuetoli*
Neapolitan	*Spuonelo* (plural: *spuonnele/spuonele*)
	*Spuònnolo*
Salento	*Ostrica rossa* (plural: *ostriche russe*)
Taranto	*Scatascuènsele*
	*Scatapunzolo*
Trieste	*Gàidero*
	*Ostrega ghèdero*

Early naturalist accounts, including those by Olivi (1792) and Chiereghin (written in 1802 and published in 2002), describe *S. gaederopus* as inhabiting the rocky substrates of the northern Adriatic Sea, particularly near Chioggia (Veneto). These records characterize the species as edible but uncommon, firmly adhering to limestone rocks at moderate depths. By the nineteenth century, *S. gaederopus* appeared sporadically in regional faunal catalogues and fishery reports. In Trieste, it was occasionally sold in local fish markets under the vernacular names *‘gàidero’* or *‘ostrica ghèdero’*, though it was rarely marketed [30,33]. Further south, the species was reported in abundance in the Gulf of Taranto [34] and the Gulf of Naples [35], where fishermen distinguished between spiny (*pungenti*) and smooth (*inermi*) forms. Local names for the mollusc included *‘scatapunzolo’* in Taranto [34] and *’*s*puonelo’* or *’spuònnolo’* (plural: *’spuonnele’*) in Neapolitan dialect [30,34,36]. Additionally, records document the species along the coasts of Sicily [37,38], with reports from Giardini noting the harvest of notably large specimens, though specific size were not provided [39]. The diversity of regional denominations and the recurrent mention of *S. gaederopus* in naturalistic literature suggests it held regional economic and long-standing cultural significance, even if its exploitation remained localized.

Although assessments of the culinary value of *S. gaederopus* were rare, the available commentary about opinions varied. The abbot Conti praised it as superior to *Ostrea edulis* [40], whereas Rondelet, quoted in Dorotea (1863), dismissed it as unpalatable:

*“* **Spuonnolo. *Spondilo comune.* Spondylus gaideropus. *Lin.***

*Abbonda in tutto il Tirreno […].*

*I pescatori napolitani distinguono gli spondili in* pungenti*, e* forti*, per essere echinati i primi, inermi i secondi.*

*Il celebre Rondelet li calunniò come quelli che prestano carni insoavi, ingrate e virose […].”*

*“* **Spuonnolo*. Common spondylus.* Spondylus gaederopus. *Lin.***

*Abundant throughout the Tyrrhenian Sea […].*

*Neapolitan fishermen distinguish spondylus into* sharp *and* strong*, the former being spiny, the latter harmless.*

*The famous Rondelet slandered them as those which provide unpleasant, disagreeable and poisonous flesh […].”*

An explicit cultural reference to *S. gaederopus* appears in the Neapolitan comic opera *Atrabilare, Commedia lirica in quattro atti*, performed at the Teatro Nuovo (Naples) in 1856 and set in Posillipo around 1760 (Fig 7). In *Act III, Scene IV*, street vendors advertise their seafood mentioning *‘spuonele’*, one of the local names for *S. gaederopus* (Table 2). This reference illustrates the species’ integration into Neapolitan daily life and its recognition in popular culture during the eighteenth and nineteenth centuries.

**Fig 7:**
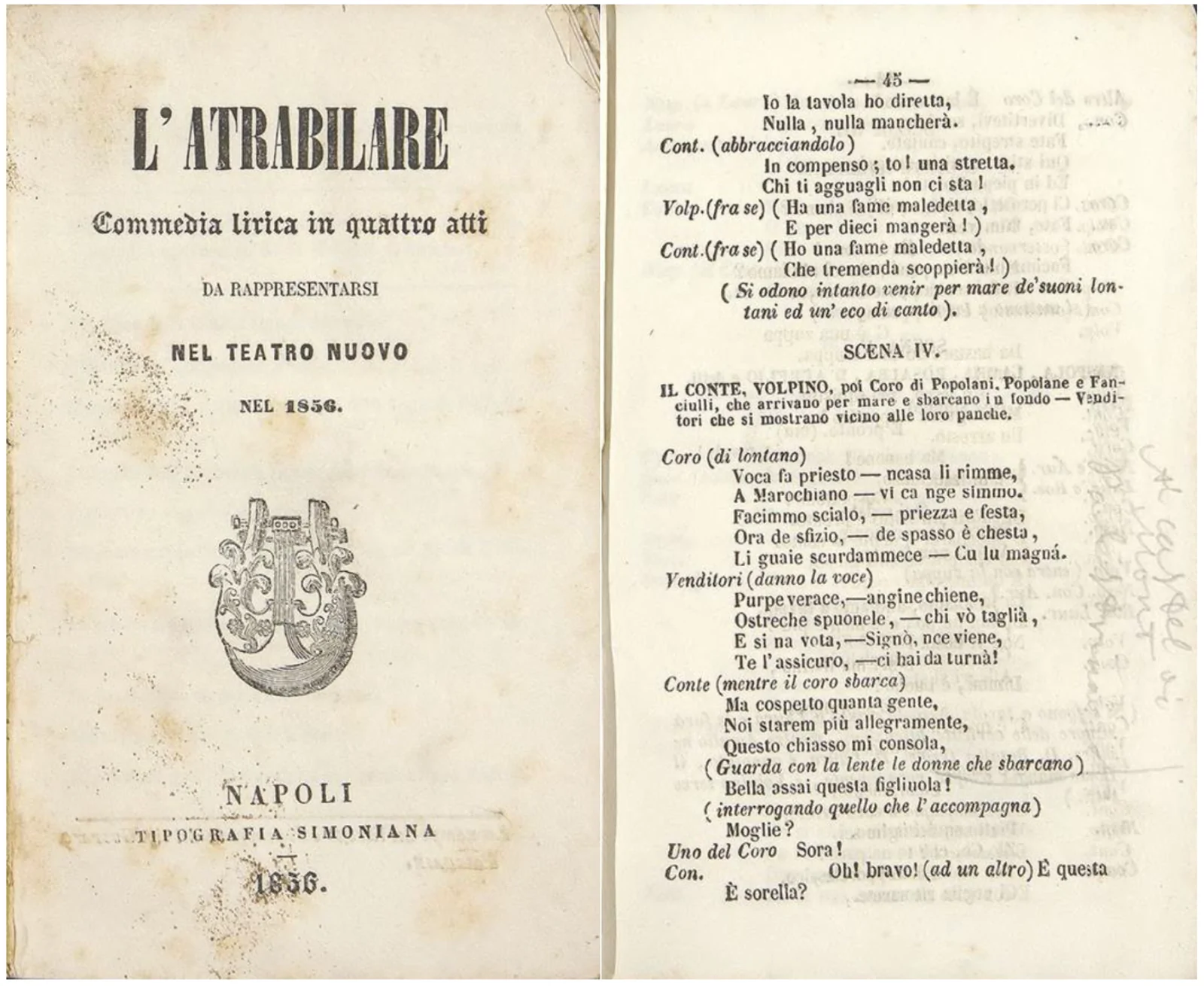
Excerpt from Atrabilare, Commedia lirica in quattro atti (Teatro Nuovo, Naples, 1856). In Atto III, Scena IV, street vendors call out seafood products, including the term ’Ostreche spuonnele’, a Neapolitan vernacular name for *Spondylus gaederopus*.

#### Local knowledge.

Today, the use of *S. gaederopus* remains extremely limited across the Mediterranean Sea, with only a few contemporary references suggesting minor commercial interest [41,42]. In Italian fishery regulations, formal recognition of *S. gaederopus* is absent [43], but the species has recently re-emerged in social medias under the name *‘ostrica imperiale’* (“imperial oyster”). Particularly along the Salento coast in Apulia (southern Italy), it has been promoted in popular media as an prized Ionian Sea delicacy, occasionally featured in coastal restaurants (e.g., Porto Cesareo [44], Bari [45], Otranto [46], or Gallipoli [47]). On social media platforms – especially among recreational fishermen and amateur gourmets – *S. gaederopus* is portrayed as a rare treasure of the sea, valued both for its flavour and the challenge of harvesting it (see search words *‘ostrica imperiale’* or *‘ostriche imperiali’* on Facebook.com). Some reports indicate prices reaching approximatively 50€ per kg in Apulian fish markets.

Local testimonies shared online by Salento fishers suggest that *S. gaederopus* populations historically formed dense shell banks, which suffered marked demographic fluctuations, including a near-collapse in the late 1970s followed by partial recovery in the following decade (Facebook.com, see comments on *S. gaederopus* pictures). Although now relatively scarce, skilled divers reportedly still collect up to 10 kg per day. Its culinary appeal remains strong, with consumption typically involving raw preparation, described as sweet and delicate.

#### Oral testimony.

An interview with an octogenarian Neapolitan recreational fisherman provided insights into the historical presence and harvesting practices of *S. gaederopus* (*’spuonelo’*) along the coast of Naples in the late 1950s and early 1960s. The informant recalled collecting the species around the age of 15 (ca. 1959–1962) in the coastal waters near Gaiola Island. Harvesting was carried out from a small wooden rowboat, using simple equipment (mask, knife, and net), with dives reaching 2 m depth. According to his account, *S. gaederopus* seldomly occurred attached to rocks, often together with large mussels (*Mytilus galloprovincialis*, ‘*cozze*’) and sea figs (*M. sabatieri*, ‘*carnummole*’), and were collected in moderate abundance (around 10–20 individuals per diving session) during the warm season, from approximately April through September. Individuals were generally small (5–6 cm, less than the size of a hand), and typically consumed raw with lemon, described as similar in taste to mussels but less sweet. The fisherman emphasized that *S. gaederopus* was rarely seen for sale, except occasionally at the fishmonger stalls (*ostricaro fisico*) in Mergellina about 65–70 years ago.

This account is particularly notable as it suggests that, even in recent history, the species was accessible at shallow depths, likely reflecting its vertical distribution since prehistory. During this earlier period, free-diving would have been the primary method of collection, as documented for ancient Mediterranean fishermen [48].

## Discussion

By integrating Early Modern paintings, archival records, and oral testimonies, this study reconstructs the overlooked ecological and cultural history of the thorny oyster (*S. gaederopus*). Despite frequent misidentification, the species appears regularly in sixteenth- to eighteenth-century Italian still-life paintings and is subsequently documented in eighteenth- and nineteenth-century naturalist texts, indicating that it was once abundant along rocky coasts of the central Mediterranean. Mid-twentieth-century oral and local accounts from southern Italy further confirm its persistence in shallow subtidal habitats and its artisanal collection using simple tools. Today, however, *S. gaederopus* is far rarer across the Mediterranean, and its population dynamics remain insufficiently documented. These findings demonstrate that historical and cultural materials can serve as valuable ecological archives, offering insights into long-term human-animal interactions and the trajectories of species that have largely disappeared from contemporary memory.

### Historical and cultural significance of *S. gaederopus*

Analysis of sixteenth- to eighteenth-century Italian still-life paintings reveals that *S. gaederopus* occupied a discreet yet significant place in Early Modern Mediterranean culture, particularly in the Campania region. The species, confidently identified by taxonomists in 43 Neapolitan artworks, most produced by members of the Recco family, was rendered with high morphological accuracy alongside other Mediterranean taxa such as sea urchins, lobsters, and goatfishes. Renowned for their still-lifes, the Recco painters frequently depicted seafood, as well as floral and vegetable arrangements. Thorny oysters appear in about one quarter of their fish still-lifes, and although fewer works by other artists were available for analysis, up to two thirds of these also feature the species. These compositions most likely depicted authentic market or coastal assemblages rather than purely symbolic scenes [25,26], and therefore provide credible visual evidence of species occurrence and human use during this period.

However, the ecological interpretation of *S. gaederopus* in historical contexts has been complicated by persistent misidentifications in art historical records. The species is never explicitly referenced in painting titles and is frequently miscatalogued as common oysters or scallops. For instance, in Giovanni Battista Recco’s *Nature morte aux poissons de mer et aux huîtres orientales* (Fig 3C), specimens of *S. gaederopus* were incorrectly identified as the great scallop (*Pecten maximus*), a species absent from the Mediterranean and restricted to the Northeast Atlantic. While the great Mediterranean scallop (*Pecten jacobaeus*) might seem a more plausible candidate, its symmetrical, fan-shaped shell bears little resemblance to the irregular, spiny morphology characteristic of *S. gaederopus*. To date, only one recent art historical study has accurately identified the species in a painting by Giovan Battista Ruoppolo [49]. Correcting this taxonomic oversight not only recovers traces of a species now rare or locally extirpated in parts of the Mediterranean but also enriches interpretations of historical biodiversity and human-nature relationships.

While archaeological evidence primarily associates *Spondylus* shells with ornamental and symbolic uses in prehistoric contexts [2,4,6], there remains a notable paucity of information regarding the species’ role during the intervening millennia, spanning antiquity and the medieval period. This gap obscures our understanding of how its cultural and economic significance may have evolved over time. By the Early Modern period, however, visual and textual records re-emphasise *S. gaederopus* as a food resource. Still-life paintings consistently depict open shells containing the organism, arranged alongside edible marine species – such as half-cut sea urchins, oysters, and razor shells – evoking scenes of market stalls or domestic kitchens. Vernacular names, including *‘spuonelo’*, *‘ostrica imperiale’*, and *‘ostrica russa’*, preserved in sources from the Modern period [30,34,36] further attest to the species’ enduring culinary and linguistic presence in Italian regional societies. Collectively, these records suggest that *S. gaederopus* held a dual role within Mediterranean cultures: as a species once valued primarily for its decorative and symbolic qualities in prehistoric times and as a culinary resource in later periods, though the processes and timing of this shift remain poorly understood due to the scarcity of evidence from the intervening centuries.

### Contemporary relevance and conservation challenges

The visibility of *S. gaederopus* appears to have declined markedly during the twentieth- and twenty-first centuries, leading to its near disappearance from cultural memory. This trend likely reflects a combination of ecological scarcity and shifting culinary preferences. In contemporary Italy, the species persists only as a fading cultural reference and a localised delicacy. In Naples, it has been largely forgotten, surviving mainly in the recollections of older fishermen who recall its occasional presence on rocky shores. By contrast, along the Salento coast (Apulia), the so-called *’ostrica imperiale’* has recently emerged in restaurant promotions on social media, where it is marketed as a rare and prestigious Ionian seafood. This limited revival may reflect both the ecological persistence of remnant populations and the enduring symbolic value of the species. Nevertheless, its exploitation remains sporadic and largely informal, constrained by harvesting difficulty, restricted distribution, and the absence of formal management or regulatory recognition [43].

Reliable scientific monitoring of population trends for *S. gaederopus* is lacking, making it difficult to assess its current status or the magnitude of its potential decline. However, comparable patterns of depletion linked to human pressures have been documented for other Mediterranean molluscs historically valued for food or ornamentation. For example, the noble pen shell (*Pinna nobilis*), once extensively harvested for its byssus fibres and edible flesh [50,51], has undergone a near-collapse, with populations almost disappearing following widespread habitat degradation and mass mortality linked to pathogenic outbreaks [52]. Similarly, intensive harvesting of the limpet *Patella ferruginea* for consumption and fishing bait has resulted in severe population declines in the western Mediterranean basin [53]. Despite strict legal protection across their biogeographic range, both species remain critically endangered [53,54].

Morphometric estimations of molluscs depicted in paintings provide approximate reference points for the size of commercially exploited seafood. Specimens attributed to *Patella* sp., for example, were estimated to reach up to 7 cm in length in the paintings examined. Although species-level identification was not possible, the observed size range (4–7 cm) broadly aligns with values reported for *Patella ferruginea* in recent studies, albeit tending toward the larger measurements recorded in protected areas, where harvesting restrictions allow larger individuals to persist, unlike in unprotected areas where large specimens are often illegally collected [53,55]. Comparisons between Early Modern Neapolitan depictions and contemporary photographs of *S. gaederopus* from southern Italy also suggest possible historical change in body size. The mean shell length of harvested individuals appears to have declined from approximately two centimetres in seventeenth-century paintings compared to present-day observations. Such patterns are consistent with well-documented ecological trends in which intensified harvesting preferentially removes large, reproductively mature individuals from mollusc populations [53,56,57].

By contrast, measurements of other molluscan taxa, including *Ostrea* sp., *Ensis siliqua*, and *Lithophaga lithophaga*, did not reveal clear size difference between historical representations and modern datasets [58,59]. These comparisons are nevertheless subject to substantial methodological limitations that constrain their value for inferring historical exploitation pressure. Measurements derived from paintings depend on indirect scaling, assumptions about perspective, and the accuracy of artistic rendering, which was primarily guided by aesthetic rather than anatomical considerations. In addition, variations in composition, restoration history, and digitization quality may alter apparent dimensions, while the absence of standardized reference objects limits the precision and reproducibility of size estimates. To improve the reliability of size analyses based on historical artworks, future research should expand the number of measurable specimens across a broader corpus of paintings and integrate modern morphometric datasets from geographically comparable regions. Despite these limitations, the observed differences remain consistent with the potential effects of size-selective harvesting of *S. gaederopus*, as well as broader environmental or biological stressors. When considered alongside testimonies, such as the one from the interviewed Neapolitan fisherman, these findings suggest that long-term exploitation may have displaced larger *S. gaederopus* individuals to deeper waters, reducing their accessibility compared to historical periods.

Taken together, these observations illustrate how cultural heritage can revive interest in historically significant marine species while simultaneously highlighting gaps in ecological knowledge. In the absence of systematic monitoring and managements frameworks, even low-intensity or niche exploitation may pose risks. This is particularly critical where species distribution, population structure, and conservation status remain poorly understood, potentially accelerating local decline and obscuring wider ecosystem vulnerability.

### Methodological contribution and future perspectives

This study highlights the methodological value of combining visual arts, historical archives, and scientific data to advance historical ecology. Still-life paintings, when examined systematically, can provide unusually rich ecological information: they document species morphology with surprising accuracy, depict associated fauna and fishing gear, and often reveal patterns of human consumption, trade, and symbolism. When these artistic sources are integrated with written archives, natural history collections, and oral testimonies, they allow for a more comprehensive reconstruction of long-term ecological change, particularly for species that are sparsely represented in conventional scientific records. Similar interdisciplinary frameworks have been increasingly recognized for their ability to reconstruct environmental histories, identify historical species distribution, and recover the ecological narratives of “forgotten” or culturally marginal species [24,25,60].

At the same time, several methodological constraints must be considered. Artistic representations may prioritize aesthetic composition over ecological accuracy, and measurements inferred from paintings are inherently approximate due to perspective, stylization, and the absence of standardized scales. Documentary and oral sources can be temporally uneven, geographically incomplete, or shaped by cultural biases in what observers considered noteworthy. Despite these limitations, the convergence of multiple independent lines of evidence demonstrates the robustness of the interdisciplinary approach. Integrating unconventional sources such as visual art into marine ecological research enriches our understanding of historical species dynamics and opens promising avenues for reconstructing past ecosystems with greater nuance and temporal depth.

## Conclusion

The thorny oyster emerges as both a historically significant species and a cultural marker of Mediterranean identity. Its contemporary decline reflects ecological pressures and changing culinary traditions, while its partial local recurrence highlights resilience and collective cultural memory. These findings emphasize the importance of integrating art and historical narratives into biodiversity research, providing a framework for recovering other “forgotten” species and enriching conservation strategies with cultural heritage dimensions.

This study highlights the value of unconventional archives, such as visual art, historical documents, and oral testimonies, in complementing ecological and conservation research. Still-life paintings, when analysed alongside historical archives and contemporary ecological data, can provide detailed insights into community composition. Incorporating these sources into historical ecology and conservation policies not only helps recover lost biodiversity baselines but also bridges cultural heritage with present-day environmental awareness. Engaging local communities and amateur naturalists through oral testimonies, participatory mapping, or digital platforms, further strengthens this interdisciplinary approach, fostering a participatory model for documenting and protecting marine biodiversity.

## Supporting information

S1 Table Catalogue of artworks analysed in this study. Overview of the artworks analysed in this study, including the artists’ names with birth and death dates, titles of the paintings, dates of production and current ownership. Paintings representing thorny oyster (*Spondylus gaederopus*) are indicated with an asterisk (*).
(XLSX)

S2 Table Taxonomic dataset of aquatic organisms depicted in the artworks. Dataset of the artworks analysed in this study, including identified aquatic taxa, the artists’ names with birth and death dates, dates of production, titles of the paintings, city of production, and current ownership.
(XLSX)

S3 Table Shell-length measurements of molluscs depicted in historical paintings and modern photographs. Measured shell lengths of molluscs (in cm) in historical paintings and modern photographs. The measurements were done with reference scale available within each image using the software ImageJ. Measurements were done on paintings P0091, P0119, P0253, P0278, P0300, P0313, P0315, P0396, P0536, P0551, P0553, P0559, P0561, P0566, P0569, P0570, P0577, P0595, P0599 and on 13 photographs found on Facebook.com.
(XLSX)

S1 Fig Number of *Spondylus gaederopus* specimen depicted in Italian still-life paintings. *S. gaederopus* depicted by Italian artists in still-life paintings produced between 1635 and 1750. Each dot corresponds to a painting.
(PDF)
