# Supplementary material for: Hidden in historical sources: Rediscovering the thorny oyster (Spondylus gaederopus Linnaeus, 1758) in art, archives, and cultural knowledge
Source: PLoS One. 2026 Oct 14;21(10):e0356273. doi: 10.1371/journal.pone.0356273 (PMC13667831; doi:10.1371/journal.pone.0356273)

Hidden in historical sources: Rediscovering the thorny oyster (*Spondylus gaederopus* Linnaeus, 1758) in art, archives, and cultural knowledge

Louise Merquiol[1,2], Anne-Sophie Tribot[2], Daniel Faget[2], Thomas Changeux[1*]

[1]Aix-Marseille Univ, Université de Toulon, CNRS, IRD, MIO, Campus Technologique et Scientifique de Luminy, 163 avenue de Luminy - Bâtiment Méditerranée, 13288 Marseille, France

[2]Aix-Marseille Univ, CNRS, TELEMME, Maison Méditerranéenne des Sciences Humaines et sociales, 5 rue Château de l'Horloge, 13090 Aix-en-Provence, France

* Corresponding author

S1 Fig. Number of *Spondylus gaederopus* specimen depicted in Italian still-life paintings. *S. gaederopus* depicted by Italian artists in still-life paintings produced between 1635 and 1750. Each dot corresponds to a painting.

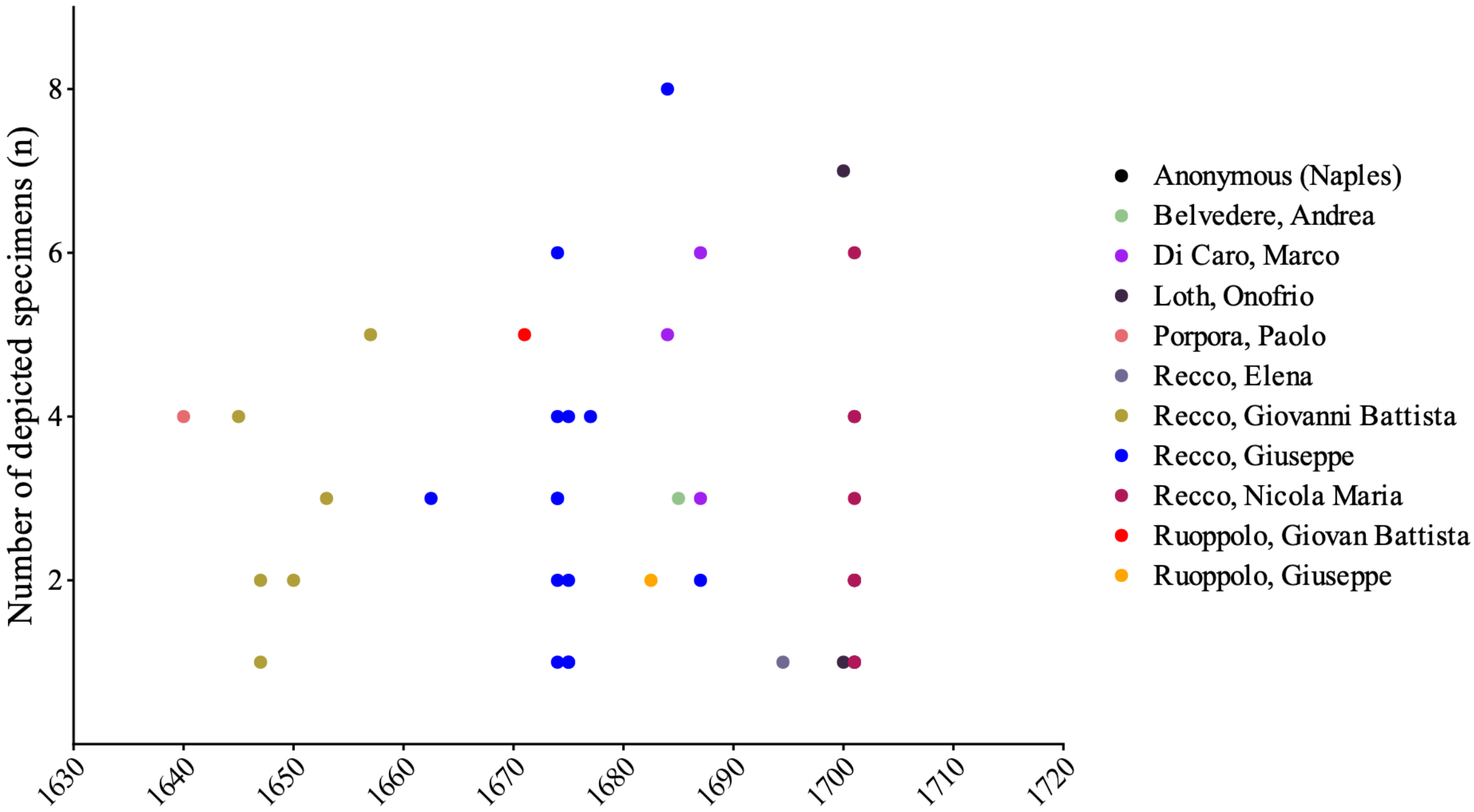

Supplement: Supplementary file 1 [file pone.0356273.s004.pdf]
