# Supplementary material for: Hidden in historical sources: Rediscovering the thorny oyster (Spondylus gaederopus Linnaeus, 1758) in art, archives, and cultural knowledge
Source: PLoS One. 2026 Oct 14;21(10):e0356273. doi: 10.1371/journal.pone.0356273 (PMC13667831; doi:10.1371/journal.pone.0356273)
Supplement: Supplementary file 2 [file pone.0356273.s006.docx]

**Response to Reviewers**

**Manuscript Title:** Hidden in historical sources: Rediscovering the thorny oyster (*Spondylus gaederopus* Linnaeus, 1758) in art, archives, and cultural knowledge

Dear Academic Editor and Reviewers,

We would like to sincerely thank you for your careful evaluation of our manuscript and for the constructive comments provided. We are grateful for the positive assessment of the originality and interdisciplinary scope of our work, and we appreciate the suggestions that have helped us improve clarity, transparency, and methodological rigor.

We have revised the manuscript accordingly and detail below our point-by-point responses. All changes are reflected in the revised manuscript and highlighted in the version with tracked changes.

**1. Editorial Requirements**

We have carefully reviewed the manuscript to ensure full compliance with PLOS ONE formatting and style guidelines, including file naming conventions and structural requirements, in accordance with the journal templates. Figure titles have been added as recommended.

The Funding Statement has been amended as requested. The updated statement now reads: “This work was supported by the Ocean & Climate PPR, jointly led by CNRS and Ifremer, and benefited from government funding managed by the French National Research Agency under the France 2030 program (reference ANR-21-POCE-0001). The funders had no role in study design, data collection and analysis, decision to publish, or preparation of the manuscript.”

All funding information has been removed from the Acknowledgments section in compliance with journal policy. The section now contains only non-financial acknowledgments.

We confirm that all data underlying the findings are fully available in accordance with PLOS ONE’s data policy. The full dataset used in this study has been uploaded as “Supporting Information 2” and the complete morphometric measurement dataset has been uploaded as “Supporting Information 3” in the file *Supporting information.xlsx*. In-text references to Supporting Information files have been revised to ensure accuracy and consistency.

We carefully addressed the copyright concerns regarding Figures 1 and 4. The maps were generated using public-domain data sources. Appropriate statements have been added in the figure captions clarifying the use of public-domain materials. No proprietary software (e.g., Google Maps or similar copyrighted platforms) was used in the final figures.

Separate captions are now provided for each figure and supplementary information and placed at the end of the manuscript. In-text figure citations have been reviewed and adjusted for consistency.

We carefully evaluated all citation suggestions and ensured that the reference list reflects only relevant and appropriate sources. No unnecessary references were added.

The reference list has been thoroughly reviewed to ensure completeness and accuracy. No retracted articles are cited in the manuscript.

**2. Response to Reviewer #1**

We thank Reviewer #1 for the very positive assessment of our interdisciplinary approach and for recommending the manuscript for publication pending minor revisions. We respond point by point below.

\# Comment 1: Please provide in the Materials and Methods the statistical analysis used.

We have now specified the statistical analyses used in the Materials and Methods section (lines 189-191). The manuscript now clearly describes the statistical approach employed to compare shell size measurements derived from painted representations and contemporary photographic specimens.

\# Comment 2: Please provide the data on the measurements either as a table in the manuscript or in the supplementary.

We have provided the complete measurement dataset as “Supporting Information 3” (Supporting information.xlsx). This file includes all individual size measurements derived from paintings and photographs, ensuring full transparency and reproducibility.

\# Comment 3: Did you compare the size of any other species depicted in the paintings with current photographs?

We agree that this would be an interesting and potentially fruitful avenue of research. However, such an analysis would constitute a distinct research question requiring dedicated methodological development. Molluscan shells can be measured with relative confidence, particularly when painted as grouped specimens (e.g., in baskets or assemblages), which likely reflects direct observation. In contrast, other organisms such as fish may present greater scaling bias. Fish may have been sketched separately before compositional integration due to their high perishability, potentially distorting proportional accuracy. We therefore consider that morphometric analysis is currently most reliable for specimens depicted in grouped assemblages.

\# Comment 4: Did you interrogate potential biases related to the dominance of one artistic family (Recco family)?

We thank the reviewer for raising this important issue. As suggested, we have added two sentences in the Discussion (lines 397-400) acknowledging the dominance of the Recco family corpus and the high possibility of thematic specialization. We also included a brief quantitative comparison with other artists. However, the number of relevant works outside the Recco corpus remains limited, preventing robust statistical comparison. This constraint is now explicitly acknowledged in the revised text.

\# Comment 5: Are there noticeable differences in the depiction of thorny oysters between 1635-1750?

We agree that detecting diachronic variation would be highly informative. We attempted to examine temporal changes and artist-specific variation in the number of depicted specimens. However, due to the limited number of artworks and uneven temporal distribution, no clear trend could be identified (although the absence of trend may also reflect genuine stability). We have added this clarification in the Results section (lines 215-219) and provided an additional visualization as “Supporting Information 4”.

**3. Response to Reviewer #2**

We are grateful to Reviewer #2 for the thorough evaluation of our manuscript and for recognizing both the originality of the dataset and the novelty of the methodological framework.

\# Comment 1: Greater caution should be applied when discussing estimated sizes and diachronic fluctuations.

We appreciate this important remark. Although methodological biases were previously acknowledged, we agree that even greater caution was warranted in the final section. We have therefore rewritten the relevant paragraph in the Discussion (lines 464-472) to more clearly emphasize the interpretive nature of artistic proportions and the inherent uncertainties involved. We now explicitly state that morphometric values derived from paintings must be regarded as approximations rather than strictly comparable biometric data. The revised text presents the findings as suggestive and hypothesis-generating rather than definitive.

\# Comment 2: Suggestion to present the work as a Systematic Review rather than a Research Article.

We appreciate the reviewer’s thoughtful assessment of the manuscript’s scope and positioning. However, we respectfully disagree that the work would be more appropriately framed as a Systematic Review. While the article engages critically with existing scholarship on *Spondylus gaederopus*, its contribution does not lie in synthesizing previously published data in a structured review format. Rather, it advances original research on the cultural, historical, and ecological trajectories of the species in the Mediterranean, drawing on archival materials, artistic representations, vernacular terminology, and newly generated morphometric data. These materials have not previously been collated, analysed, or interpreted in relation to one another. The manuscript formulates specific research questions and provides new documentary and quantitative evidence to address them. The methodological framework is that of an original historical and ecological investigation grounded in contextual and analytical interpretation. For these reasons, we believe the article fulfils the criteria of a Research Article: it presents novel primary data, it applies an explicit analytical framework, and it advances new interpretations that extend beyond summarization of existing literature.

**Closing Statement**

We sincerely thank both reviewers and the Academic Editor for their insightful comments, which have significantly strengthened the manuscript. We believe the revisions improve methodological clarity, interpretive caution, and transparency, while reinforcing the originality and scientific contribution of the study.

We hope the revised version meets the expectations of the editor and reviewers, and we remain at your disposal for any further clarification.

With kind regards,

Thomas Changeux

on behalf of all co-authors
