# Supplementary material for: Hidden in historical sources: Rediscovering the thorny oyster (Spondylus gaederopus Linnaeus, 1758) in art, archives, and cultural knowledge
Source: PLoS One. 2026 Oct 14;21(10):e0356273. doi: 10.1371/journal.pone.0356273 (PMC13667831; doi:10.1371/journal.pone.0356273)
Supplement: Supplementary file 3 [file pone.0356273.s007.docx]

**Response to Reviewers**

**Manuscript Title:** Hidden in historical sources: Rediscovering the thorny oyster (*Spondylus gaederopus* Linnaeus, 1758) in art, archives, and cultural knowledge

Dear Academic Editor and Reviewers,

Again, we would like to sincerely thank you for your careful evaluation of our manuscript and for the constructive comments provided. We are grateful for the positive assessment of the originality and interdisciplinary scope of our work, and we appreciate the suggestions that have helped us improve clarity, transparency, and methodological rigor.

Following our revised manuscript according to the reviewers, we addressed the very last comment of Reviewer #1. All changes are reflected in the revised manuscript and highlighted in the version with tracked changes.

**2. Response to Reviewer #1**

We thank Reviewer #1 for the second review of our manuscript and their very positive assessment. We respond below to their last concern.

\# Comment 1: I have no ongoing concerns but I would like to clarify one of the questions I previously asked. My question on 'Did you compare the size of any other species depicted in the paintings with current photographs?' was more specific to the other mollusc species depicted rather than other organisms. As it gives more weight to the results if similar trends are seen with other molluscs, where a robust methodology has already been established. For example something like Mytilus which has been well studied and there is ample material out there, it would be good to see if those same trends occur.

We agree with this point regarding size estimation and have addressed it by measuring the lengths of co-occurring molluscs depicted in the paintings (lines 173-182, lines 238-242, Figure 4), and Supporting information 3). As expected, limpets (*Patella* sp.), for example, tend to appear larger in the paintings than in recent monitoring studies, although direct comparisons from the same geographic areas are not currently available. In contrast, other taxa show size ranges broadly comparable to present-day observations. This discrepancy may reflect the absence of spatially matched datasets, regional variations in growth conditions, or the limited number of measurable specimens. These constraints and their implications are now explicitly addressed in the manuscript (lines 447-461).

We hope the revised version meets the expectations of the editor and reviewers, and we remain at your disposal for any further clarification.

With kind regards,

Thomas Changeux

on behalf of all co-authors
