# Supplementary material for: Hidden in historical sources: Rediscovering the thorny oyster (Spondylus gaederopus Linnaeus, 1758) in art, archives, and cultural knowledge
Source: PLoS One. 2026 Oct 14;21(10):e0356273. doi: 10.1371/journal.pone.0356273 (PMC13667831; doi:10.1371/journal.pone.0356273)
Supplement: Peer review history

# Peer review: Decision Letter 0

**DOI:** 10.1371/journal.pone.0356273.r001

**Authors:** José A. Fernández Robledo

18 Feb 2026

Dear Dr. Changeux,

Thank you for submitting your manuscript to PLOS ONE. After careful consideration, we feel that it has merit but does not fully meet PLOS ONE’s publication criteria as it currently stands. Therefore, we invite you to submit a revised version of the manuscript that addresses the points raised during the review process.

Dear Dr. Changeux

One of the reviewers has minor comments. Please address them in the reviewed version.

Thank you,

Please submit your revised manuscript by Apr 04 2026 11:59PM. If you will need more time than this to complete your revisions, please reply to this message or contact the journal office at plosone@plos.org. When you're ready to submit your revision, log on to https://www.editorialmanager.com/pone/ and select the 'Submissions Needing Revision' folder to locate your manuscript file.

- A letter that responds to each point raised by the academic editor and reviewer(s). You should upload this letter as a separate file labeled 'Response to Reviewers'.
- A marked-up copy of your manuscript that highlights changes made to the original version. You should upload this as a separate file labeled 'Revised Manuscript with Track Changes'.
- An unmarked version of your revised paper without tracked changes. You should upload this as a separate file labeled 'Manuscript'.

If applicable, we recommend that you deposit your laboratory protocols in protocols.io to enhance the reproducibility of your results. Protocols.io assigns your protocol its own identifier (DOI) so that it can be cited independently in the future. For instructions see: https://journals.plos.org/plosone/s/submission-guidelines#loc-laboratory-protocols. Additionally, PLOS ONE offers an option for publishing peer-reviewed Lab Protocol articles, which describe protocols hosted on protocols.io. Read more information on sharing protocols at https://plos.org/protocols?utm_medium=editorial-email&utm_source=authorletters&utm_campaign=protocols.

We look forward to receiving your revised manuscript.

Kind regards,

José A. Fernández Robledo, Ph.D.

Academic Editor

PLOS One

Journal Requirements:

When submitting your revision, we need you to address these additional requirements.

1. Please ensure that your manuscript meets PLOS ONE's style requirements, including those for file naming. The PLOS ONE style templates can be found at

https://journals.plos.org/plosone/s/file?id=wjVg/PLOSOne_formatting_sample_main_body.pdf and

https://journals.plos.org/plosone/s/file?id=ba62/PLOSOne_formatting_sample_title_authors_affiliations.pdf

2.  Thank you for stating the following financial disclosure:

“This work was supported by the Ocean & Climate PPR, jointly led by CNRS and Ifremer, and benefited from government funding managed by the French National Research Agency under the France 2030 program (reference ANR-21-POCE-0001). “

Please state what role the funders took in the study. If the funders had no role, please state: "The funders had no role in study design, data collection and analysis, decision to publish, or preparation of the manuscript."

If this statement is not correct you must amend it as needed.

Please include this amended Role of Funder statement in your cover letter; we will change the online submission form on your behalf.

3. Thank you for stating the following in the Acknowledgments Section of your manuscript:

“This work was supported by the Ocean & Climate PPR, jointly led by CNRS and Ifremer,  and benefited from government funding managed by the French National Research Agency under the France 2030 program (reference ANR-21-POCE-0001). We thank Philippe Béarez 5(MNHN, France), Julio Cotom (MNHN, France), and Vianney Forest (Inrap, France) for their assistance in identifying S. gaederopus in the paintings. We are also highly grateful to Maria Lucia De Nicolò (Università degli Studi di Bologna, Italy) for her valuable historical contributions, and to Tatiana Theodoropoulou (CNRS, France) for her archaeological insights. Finally, we thank Gennaro Galario, the Neapolitan recreational fishermen  interviewed for this study, for sharing his oral testimony.”

We note that you have provided funding information that is not currently declared in your Funding Statement. However, funding information should not appear in the Acknowledgments section or other areas of your manuscript. We will only publish funding information present in the Funding Statement section of the online submission form.

Please remove any funding-related text from the manuscript and let us know how you would like to update your Funding Statement. Currently, your Funding Statement reads as follows:

“This work was supported by the Ocean & Climate PPR, jointly led by CNRS and Ifremer, and benefited from government funding managed by the French National Research Agency under the France 2030 program (reference ANR-21-POCE-0001). “

Please include your amended statements within your cover letter; we will change the online submission form on your behalf.

4. In the online submission form, you indicated that “The data are contained within the manuscript and/or Supporting Information files. If it is not the case they are available on request to the corresponding author.”

All PLOS journals now require all data underlying the findings described in their manuscript to be freely available to other researchers, either 1. In a public repository, 2. Within the manuscript itself, or 3. Uploaded as supplementary information.

This policy applies to all data except where public deposition would breach compliance with the protocol approved by your research ethics board. If your data cannot be made publicly available for ethical or legal reasons (e.g., public availability would compromise patient privacy), please explain your reasons on resubmission and your exemption request will be escalated for approval.

5. We note that Figure 1 and 4 in your submission contain [map/satellite] images which may be copyrighted. All PLOS content is published under the Creative Commons Attribution License (CC BY 4.0), which means that the manuscript, images, and Supporting Information files will be freely available online, and any third party is permitted to access, download, copy, distribute, and use these materials in any way, even commercially, with proper attribution. For these reasons, we cannot publish previously copyrighted maps or satellite images created using proprietary data, such as Google software (Google Maps, Street View, and Earth). For more information, see our copyright guidelines: http://journals.plos.org/plosone/s/licenses-and-copyright.

We require you to either (1) present written permission from the copyright holder to publish these figures specifically under the CC BY 4.0 license, or (2) remove the figures from your submission:

1. You may seek permission from the original copyright holder of Figure 1 and 4 to publish the content specifically under the CC BY 4.0 license.

We recommend that you contact the original copyright holder with the Content Permission Form (http://journals.plos.org/plosone/s/file?id=7c09/content-permission-form.pdf) and the following text:

“I request permission for the open-access journal PLOS ONE to publish XXX under the Creative Commons Attribution License (CCAL) CC BY 4.0 (http://creativecommons.org/licenses/by/4.0/). Please be aware that this license allows unrestricted use and distribution, even commercially, by third parties. Please reply and provide explicit written permission to publish XXX under a CC BY license and complete the attached form.”

Please upload the completed Content Permission Form or other proof of granted permissions as an ""Other"" file with your submission.

In the figure caption of the copyrighted figure, please include the following text: “Reprinted from [ref] under a CC BY license, with permission from [name of publisher], original copyright [original copyright year].”

2. If you are unable to obtain permission from the original copyright holder to publish these figures under the CC BY 4.0 license or if the copyright holder’s requirements are incompatible with the CC BY 4.0 license, please either i) remove the figure or ii) supply a replacement figure that complies with the CC BY 4.0 license. Please check copyright information on all replacement figures and update the figure caption with source information. If applicable, please specify in the figure caption text when a figure is similar but not identical to the original image and is therefore for illustrative purposes only.

The following resources for replacing copyrighted map figures may be helpful:

USGS National Map Viewer (public domain): http://viewer.nationalmap.gov/viewer/

The Gateway to Astronaut Photography of Earth (public domain): http://eol.jsc.nasa.gov/sseop/clickmap/

Maps at the CIA (public domain): https://www.cia.gov/library/publications/the-world-factbook/index.html and https://www.cia.gov/library/publications/cia-maps-publications/index.html

NASA Earth Observatory (public domain): http://earthobservatory.nasa.gov/

Landsat: http://landsat.visibleearth.nasa.gov/

USGS EROS (Earth Resources Observatory and Science (EROS) Center) (public domain): http://eros.usgs.gov/#

Natural Earth (public domain): http://www.naturalearthdata.com/

6. Please include a separate caption for each figure in your manuscript.

7. Please include captions for your Supporting Information files at the end of your manuscript, and update any in-text citations to match accordingly. Please see our Supporting Information guidelines for more information: http://journals.plos.org/plosone/s/supporting-information.

8. If the reviewer comments include a recommendation to cite specific previously published works, please review and evaluate these publications to determine whether they are relevant and should be cited. There is no requirement to cite these works unless the editor has indicated otherwise.

9. Please review your reference list to ensure that it is complete and correct. If you have cited papers that have been retracted, please include the rationale for doing so in the manuscript text, or remove these references and replace them with relevant current references. Any changes to the reference list should be mentioned in the rebuttal letter that accompanies your revised manuscript. If you need to cite a retracted article, indicate the article’s retracted status in the References list and also include a citation and full reference for the retraction notice.

[Note: HTML markup is below. Please do not edit.]

Reviewers' comments:

Reviewer's Responses to Questions

**Comments to the Author**

1. Is the manuscript technically sound, and do the data support the conclusions?

Reviewer #1: Yes

Reviewer #2: Yes

**********

2. Has the statistical analysis been performed appropriately and rigorously?

Reviewer #1: I Don't Know

Reviewer #2: Yes

**********

3. Have the authors made all data underlying the findings in their manuscript fully available?

The PLOS Data policy

Reviewer #1: No

Reviewer #2: Yes

**********

4. Is the manuscript presented in an intelligible fashion and written in standard English?

Reviewer #1: Yes

Reviewer #2: Yes

**********

Reviewer #1: The authors here have used an interdisciplinary approach to occurrence and human use of thorny oysters throughout history. It is a really interesting unique study which uses art historical analysis, taxonomy, archival research and ethnobiological investigation. The authors show that this type of interdisciplinary research is extremely useful in understanding population dynamics and human use of bivalve species. I recommend this paper to be accepted after a few minor corrections.

1) Please provide in the material and methods the statistical analysis used

2) Please provide the data on the measurements either as a table in the manuscript or in the supplementary.

These are just my other thoughts to consider:

1) Did you compare the size of any other species depicted in the paintings with current photographs? It would be interesting to see if the same trend is apparent, especially if it is a species where there is more information on abundance and distribution through time, just to use as confirmation.

2) Did you interrogate the biases involved with the family? As the majority of paintings are by one family it would be interesting to assess the biases, did they have a preference for depicting images with seafood and specifically the thorny oyster? What is the difference in the depiction of thorny oysters between the family and other artists? Additionally, do you notice a difference in the depiction of the thorny oysters across the time of the paintings 1635-1750? It would be interesting to know if over that short period of time, a noticeable difference was apparent.

Reviewer #2: The manuscript presents an original and compelling interdisciplinary approach to reconstructing the historical ecology of the Mediterranean thorny oyster (Spondylus gaederopus). The integration of art history, archival research, and ethnobiological data is well-executed and provides new insights into the long-term demographic dynamics and human exploitation of this species. The methodology represents a novel approach and the authors carefully address potential biases inherent in using iconographic sources for scientific data. The text is clear and the English form good.

The authors' generation of primary quantitative data (e.g., the comparative shell size analysis between historical paintings and modern specimens) and the application of a novel methodological framework to a previously unanalysed dataset of Italian still-life paintings is very peculiar and interesting for the scientific community. However, I have just one concerns regarding this manuscript. Even though, the work goes beyond synthesizing existing knowledge and provides potential evidence for a decline in the species' size over centuries, the authors should discuss the 'estimated sizes' of the individuals and their diachronic fluctuations with even greater caution in the final section of the text, given the inherently interpretive nature of artistic proportions.

Furthermore, I have some reservations regarding whether the manuscript possesses sufficient scientific weight to be accepted as a Research Article; I would instead suggest presenting this work as a Systematic Review.

**********

what does this mean?). If published, this will include your full peer review and any attached files.

If you choose “no”, your identity will remain anonymous but your review may still be made public.

**Do you want your identity to be public for this peer review?**  For information about this choice, including consent withdrawal, please see our Privacy Policy

Reviewer #1: No

Reviewer #2: No

**********

[NOTE: If reviewer comments were submitted as an attachment file, they will be attached to this email and accessible via the submission site. Please log into your account, locate the manuscript record, and check for the action link "View Attachments". If this link does not appear, there are no attachment files.]

To ensure your figures meet our technical requirements, please review our figure guidelines: https://journals.plos.org/plosone/s/figures

You may also use PLOS’s free figure tool, NAAS, to help you prepare publication quality figures: https://journals.plos.org/plosone/s/figures#loc-tools-for-figure-preparation.

NAAS will assess whether your figures meet our technical requirements by comparing each figure against our figure specifications.

# Author comment: Author response to Decision Letter 1

**DOI:** 10.1371/journal.pone.0356273.r002

6 Mar 2026

Response to Reviewers

Manuscript Title: Hidden in historical sources: Rediscovering the thorny oyster (Spondylus gaederopus Linnaeus, 1758) in art, archives, and cultural knowledge

Dear Academic Editor and Reviewers,

We would like to sincerely thank you for your careful evaluation of our manuscript and for the constructive comments provided. We are grateful for the positive assessment of the originality and interdisciplinary scope of our work, and we appreciate the suggestions that have helped us improve clarity, transparency, and methodological rigor.

We have revised the manuscript accordingly and detail below our point-by-point responses. All changes are reflected in the revised manuscript and highlighted in the version with tracked changes.

1. Editorial Requirements

We have carefully reviewed the manuscript to ensure full compliance with PLOS ONE formatting and style guidelines, including file naming conventions and structural requirements, in accordance with the journal templates. Figure titles have been added as recommended.

The Funding Statement has been amended as requested. The updated statement now reads: “This work was supported by the Ocean & Climate PPR, jointly led by CNRS and Ifremer, and benefited from government funding managed by the French National Research Agency under the France 2030 program (reference ANR-21-POCE-0001). The funders had no role in study design, data collection and analysis, decision to publish, or preparation of the manuscript.”

All funding information has been removed from the Acknowledgments section in compliance with journal policy. The section now contains only non-financial acknowledgments.

We confirm that all data underlying the findings are fully available in accordance with PLOS ONE’s data policy. The full dataset used in this study has been uploaded as “Supporting Information 2” and the complete morphometric measurement dataset has been uploaded as “Supporting Information 3” in the file Supporting information.xlsx. In-text references to Supporting Information files have been revised to ensure accuracy and consistency.

We carefully addressed the copyright concerns regarding Figures 1 and 4. The maps were generated using public-domain data sources. Appropriate statements have been added in the figure captions clarifying the use of public-domain materials. No proprietary software (e.g., Google Maps or similar copyrighted platforms) was used in the final figures.

Separate captions are now provided for each figure and supplementary information and placed at the end of the manuscript. In-text figure citations have been reviewed and adjusted for consistency.

We carefully evaluated all citation suggestions and ensured that the reference list reflects only relevant and appropriate sources. No unnecessary references were added.

The reference list has been thoroughly reviewed to ensure completeness and accuracy. No retracted articles are cited in the manuscript.

2. Response to Reviewer #1

We thank Reviewer #1 for the very positive assessment of our interdisciplinary approach and for recommending the manuscript for publication pending minor revisions. We respond point by point below.

# Comment 1: Please provide in the Materials and Methods the statistical analysis used.

We have now specified the statistical analyses used in the Materials and Methods section (lines 189-191). The manuscript now clearly describes the statistical approach employed to compare shell size measurements derived from painted representations and contemporary photographic specimens.

# Comment 2: Please provide the data on the measurements either as a table in the manuscript or in the supplementary.

We have provided the complete measurement dataset as “Supporting Information 3” (Supporting information.xlsx). This file includes all individual size measurements derived from paintings and photographs, ensuring full transparency and reproducibility.

# Comment 3: Did you compare the size of any other species depicted in the paintings with current photographs?

We agree that this would be an interesting and potentially fruitful avenue of research. However, such an analysis would constitute a distinct research question requiring dedicated methodological development. Molluscan shells can be measured with relative confidence, particularly when painted as grouped specimens (e.g., in baskets or assemblages), which likely reflects direct observation. In contrast, other organisms such as fish may present greater scaling bias. Fish may have been sketched separately before compositional integration due to their high perishability, potentially distorting proportional accuracy. We therefore consider that morphometric analysis is currently most reliable for specimens depicted in grouped assemblages.

# Comment 4: Did you interrogate potential biases related to the dominance of one artistic family (Recco family)?

We thank the reviewer for raising this important issue. As suggested, we have added two sentences in the Discussion (lines 397-400) acknowledging the dominance of the Recco family corpus and the high possibility of thematic specialization. We also included a brief quantitative comparison with other artists. However, the number of relevant works outside the Recco corpus remains limited, preventing robust statistical comparison. This constraint is now explicitly acknowledged in the revised text.

# Comment 5: Are there noticeable differences in the depiction of thorny oysters between 1635-1750?

We agree that detecting diachronic variation would be highly informative. We attempted to examine temporal changes and artist-specific variation in the number of depicted specimens. However, due to the limited number of artworks and uneven temporal distribution, no clear trend could be identified (although the absence of trend may also reflect genuine stability). We have added this clarification in the Results section (lines 215-219) and provided an additional visualization as “Supporting Information 4”.

3. Response to Reviewer #2

We are grateful to Reviewer #2 for the thorough evaluation of our manuscript and for recognizing both the originality of the dataset and the novelty of the methodological framework.

# Comment 1: Greater caution should be applied when discussing estimated sizes and diachronic fluctuations.

We appreciate this important remark. Although methodological biases were previously acknowledged, we agree that even greater caution was warranted in the final section. We have therefore rewritten the relevant paragraph in the Discussion (lines 464-472) to more clearly emphasize the interpretive nature of artistic proportions and the inherent uncertainties involved. We now explicitly state that morphometric values derived from paintings must be regarded as approximations rather than strictly comparable biometric data. The revised text presents the findings as suggestive and hypothesis-generating rather than definitive.

# Comment 2: Suggestion to present the work as a Systematic Review rather than a Research Article.

We appreciate the reviewer’s thoughtful assessment of the manuscript’s scope and positioning. However, we respectfully disagree that the work would be more appropriately framed as a Systematic Review. While the article engages critically with existing scholarship on Spondylus gaederopus, its contribution does not lie in synthesizing previously published data in a structured review format. Rather, it advances original research on the cultural, historical, and ecological trajectories of the species in the Mediterranean, drawing on archival materials, artistic representations, vernacular terminology, and newly generated morphometric data. These materials have not previously been collated, analysed, or interpreted in relation to one another. The manuscript formulates specific research questions and provides new documentary and quantitative evidence to address them. The methodological framework is that of an original historical and ecological investigation grounded in contextual and analytical interpretation. For these reasons, we believe the article fulfils the criteria of a Research Article: it presents novel primary data, it applies an explicit analytical framework, and it advances new interpretations that extend beyond summarization of existing literature.

Closing Statement

We sincerely thank both reviewers and the Academic Editor for their insightful comments, which have significantly strengthened the manuscript. We believe the revisions improve methodological clarity, interpretive caution, and transparency, while reinforcing the originality and scientific contribution of the study.

We hope the revised version meets the expectations of the editor and reviewers, and we remain at your disposal for any further clarification.

With kind regards,

Thomas Changeux

on behalf of all co-authors

Attachment Submitted filename: Response to reviewers.docx

# Peer review: Decision Letter 1

**DOI:** 10.1371/journal.pone.0356273.r003

**Authors:** José A. Fernández Robledo

20 Apr 2026

Dear Dr. Changeux,

Thank you for submitting your manuscript to PLOS ONE. After careful consideration, we feel that it has merit but does not fully meet PLOS ONE’s publication criteria as it currently stands. Therefore, we invite you to submit a revised version of the manuscript that addresses the points raised during the review process.

==============================

Dear Dr. Changeux.

The reviewer has "Accepted" the revised version. Still, there is a concern that the authors should be addressed before I accept the manuscript.

"I have no ongoing concerns but I would like to clarify one of the questions I previously asked. My question on 'Did you compare the size of any other species depicted in the paintings with current photographs?' was more specific to the other mollusc species depicted rather than other organisms. As it gives more weight to the results if similar trends are seen with other molluscs, where a robust methodology has already been established. For example something like Mytilus which has been well studied and there is ample material out there, it would be good to see if those same trends occur."

Sincerely,

-j

==============================

Please submit your revised manuscript by Jun 04 2026 11:59PM. If you will need more time than this to complete your revisions, please reply to this message or contact the journal office at plosone@plos.org. When you're ready to submit your revision, log on to https://www.editorialmanager.com/pone/ and select the 'Submissions Needing Revision' folder to locate your manuscript file.

- A letter that responds to each point raised by the academic editor and reviewer(s). You should upload this letter as a separate file labeled 'Response to Reviewers'.
- A marked-up copy of your manuscript that highlights changes made to the original version. You should upload this as a separate file labeled 'Revised Manuscript with Track Changes'.
- An unmarked version of your revised paper without tracked changes. You should upload this as a separate file labeled 'Manuscript'.

If you would like to make changes to your financial disclosure, please include your updated statement in your cover letter. Guidelines for resubmitting your figure files are available below the reviewer comments at the end of this letter.

If applicable, we recommend that you deposit your laboratory protocols in protocols.io to enhance the reproducibility of your results. Protocols.io assigns your protocol its own identifier (DOI) so that it can be cited independently in the future. For instructions see: https://journals.plos.org/plosone/s/submission-guidelines#loc-laboratory-protocols. Additionally, PLOS ONE offers an option for publishing peer-reviewed Lab Protocol articles, which describe protocols hosted on protocols.io. Read more information on sharing protocols at https://plos.org/protocols?utm_medium=editorial-email&utm_source=authorletters&utm_campaign=protocols.

As the corresponding author, your ORCID iD is verified in the submission system and will appear in the published article. PLOS supports the use of ORCID, and we encourage all coauthors to register for an ORCID iD and use it as well. Please encourage your coauthors to verify their ORCID iD within the submission system before final acceptance, as unverified ORCID iDs will not appear in the published article. *Only*  the individual author can complete the verification step; PLOS staff *cannot*  verify ORCID iDs on behalf of authors.

We look forward to receiving your revised manuscript.

Kind regards,

José A. Fernández Robledo, Ph.D.

Academic Editor

PLOS One

Journal Requirements:

If the reviewer comments include a recommendation to cite specific previously published works, please review and evaluate these publications to determine whether they are relevant and should be cited. There is no requirement to cite these works unless the editor has indicated otherwise.

Please review your reference list to ensure that it is complete and correct. If you have cited papers that have been retracted, please include the rationale for doing so in the manuscript text, or remove these references and replace them with relevant current references. Any changes to the reference list should be mentioned in the rebuttal letter that accompanies your revised manuscript. If you need to cite a retracted article, indicate the article’s retracted status in the References list and also include a citation and full reference for the retraction notice.

[Note: HTML markup is below. Please do not edit.]

Reviewers' comments:

Reviewer's Responses to Questions

**Comments to the Author**

Reviewer #1: All comments have been addressed

**********

2. Is the manuscript technically sound, and do the data support the conclusions?

Reviewer #1: Yes

**********

3. Has the statistical analysis been performed appropriately and rigorously?

Reviewer #1: Yes

**********

4. Have the authors made all data underlying the findings in their manuscript fully available?

The PLOS Data policy

Reviewer #1: Yes

**********

5. Is the manuscript presented in an intelligible fashion and written in standard English?

Reviewer #1: Yes

**********

Reviewer #1: I thank the authors for addressing the comments in the first review.

I have no ongoing concerns but i would like to clarify one of the questions i previously asked. My question on 'Did you compare the size of any other species depicted in the paintings with current photographs?' was more specific to the other mollusc species depicted rather than other organisms. As it gives more weight to the results if similar trends are seen with other molluscs, where a robust methodology has already been established. For example something like Mytilus which has been well studied and there is ample material out there, it would be good to see if those same trends occur.

**********

what does this mean?). If published, this will include your full peer review and any attached files.

If you choose “no”, your identity will remain anonymous but your review may still be made public.

**Do you want your identity to be public for this peer review?**  For information about this choice, including consent withdrawal, please see our Privacy Policy

Reviewer #1: No

**********

[NOTE: If reviewer comments were submitted as an attachment file, they will be attached to this email and accessible via the submission site. Please log into your account, locate the manuscript record, and check for the action link "View Attachments". If this link does not appear, there are no attachment files.]

To ensure your figures meet our technical requirements, please review our figure guidelines: https://journals.plos.org/plosone/s/figures

You may also use PLOS’s free figure tool, NAAS, to help you prepare publication quality figures: https://journals.plos.org/plosone/s/figures#loc-tools-for-figure-preparation.

NAAS will assess whether your figures meet our technical requirements by comparing each figure against our figure specifications.

# Author comment: Author response to Decision Letter 2

**DOI:** 10.1371/journal.pone.0356273.r004

9 Jul 2026

Response to Reviewers

Manuscript Title: Hidden in historical sources: Rediscovering the thorny oyster (Spondylus gaederopus Linnaeus, 1758) in art, archives, and cultural knowledge

Dear Academic Editor and Reviewers,

Again, we would like to sincerely thank you for your careful evaluation of our manuscript and for the constructive comments provided. We are grateful for the positive assessment of the originality and interdisciplinary scope of our work, and we appreciate the suggestions that have helped us improve clarity, transparency, and methodological rigor.

Following our revised manuscript according to the reviewers, we addressed the very last comment of Reviewer #1. All changes are reflected in the revised manuscript and highlighted in the version with tracked changes.

2. Response to Reviewer #1

We thank Reviewer #1 for the second review of our manuscript and their very positive assessment. We respond below to their last concern.

# Comment 1: I have no ongoing concerns but I would like to clarify one of the questions I previously asked. My question on 'Did you compare the size of any other species depicted in the paintings with current photographs?' was more specific to the other mollusc species depicted rather than other organisms. As it gives more weight to the results if similar trends are seen with other molluscs, where a robust methodology has already been established. For example something like Mytilus which has been well studied and there is ample material out there, it would be good to see if those same trends occur.

We agree with this point regarding size estimation and have addressed it by measuring the lengths of co-occurring molluscs depicted in the paintings (lines 173-182, lines 238-242, Figure 4), and Supporting information 3). As expected, limpets (Patella sp.), for example, tend to appear larger in the paintings than in recent monitoring studies, although direct comparisons from the same geographic areas are not currently available. In contrast, other taxa show size ranges broadly comparable to present-day observations. This discrepancy may reflect the absence of spatially matched datasets, regional variations in growth conditions, or the limited number of measurable specimens. These constraints and their implications are now explicitly addressed in the manuscript (lines 447-461).

We hope the revised version meets the expectations of the editor and reviewers, and we remain at your disposal for any further clarification.

With kind regards,

Thomas Changeux

on behalf of all co-authors

Attachment Submitted filename: Response to reviewers_V3.docx

# Editor report: Decision Letter 2

**DOI:** 10.1371/journal.pone.0356273.r005

**Authors:** José A. Fernández Robledo

2 Aug 2026

Hidden in historical sources: Rediscovering the thorny oyster (Spondylus gaederopus Linnaeus, 1758) in art, archives, and cultural knowledge

PONE-D-25-67470R2

Dear Dr. Changeux,

We’re pleased to inform you that your manuscript has been judged scientifically suitable for publication and will be formally accepted for publication once it meets all outstanding technical requirements.

Within one week, you’ll receive an e-mail detailing the required amendments. When these have been addressed, you’ll receive a formal acceptance letter and your manuscript will be scheduled for publication.

An invoice will be generated when your article is formally accepted. Please note, if your institution has a publishing partnership with PLOS and your article meets the relevant criteria, all or part of your publication costs will be covered. Please make sure your user information is up-to-date by logging into Editorial Manager at Editorial Manager® and clicking the ‘Update My Information' link at the top of the page. For questions related to billing, please contact billing support.

If your institution or institutions have a press office, please notify them about your upcoming paper to help maximize its impact. If they’ll be preparing press materials, please inform our press team as soon as possible -- no later than 48 hours after receiving the formal acceptance. Your manuscript will remain under strict press embargo until 2 pm Eastern Time on the date of publication. For more information, please contact onepress@plos.org.

Kind regards,

José A. Fernández Robledo, Ph.D.

Academic Editor

PLOS One

Additional Editor Comments (optional):

Reviewers' comments:

# Editor report: Acceptance letter

**DOI:** 10.1371/journal.pone.0356273.r006

**Authors:** José A. Fernández Robledo

PONE-D-25-67470R2

PLOS One

Dear Dr. Changeux,

I'm pleased to inform you that your manuscript has been deemed suitable for publication in PLOS One. Congratulations! Your manuscript is now being handed over to our production team.

At this stage, our production department will prepare your paper for publication. This includes ensuring the following:

* All references, tables, and figures are properly cited

* All relevant supporting information is included in the manuscript submission,

* There are no issues that prevent the paper from being properly typeset

You will receive further instructions from the production team, including instructions on how to review your proof when it is ready. Please keep in mind that we are working through a large volume of accepted articles, so please give us a few days to review your paper and let you know the next and final steps.

Lastly, if your institution or institutions have a press office, please let them know about your upcoming paper now to help maximize its impact. If they'll be preparing press materials, please inform our press team within the next 48 hours. Your manuscript will remain under strict press embargo until 2 pm Eastern Time on the date of publication. For more information, please contact onepress@plos.org.

You will receive an invoice from PLOS for your publication fee after your manuscript has reached the completed accept phase. If you receive an email requesting payment before acceptance or for any other service, this may be a phishing scheme. Learn how to identify phishing emails and protect your accounts at https://explore.plos.org/phishing.

If we can help with anything else, please email us at customercare@plos.org.

Thank you for submitting your work to PLOS One and supporting open access.

Kind regards,

PLOS One Editorial Office Staff

on behalf of

Dr. José A. Fernández Robledo

Academic Editor

PLOS One
